# A scoping review of innovations that promote interprofessional collaboration (IPC) in primary care for older adults living with age-related chronic disease in rural areas

**DOI:** 10.1371/journal.pone.0331327

**Published:** 2025-09-03

**Authors:** Valerie Elliot, Julie Kosteniuk, Duane P. Minish, Chelsie Cameron, Megan E. O’Connell, Debra Morgan

**Affiliations:** 1 Rural Dementia Action Research (RaDAR), Canadian Centre for Rural and Agricultural Health, University of Saskatchewan, Saskatoon, Saskatchewan, Canada; 2 Department of Psychology, University of Saskatchewan, Saskatoon, Saskatchewan, Canada; Cardiff University, UNITED KINGDOM OF GREAT BRITAIN AND NORTHERN IRELAND

## Abstract

**Background and objectives:**

An aging population and associated multi-morbid chronic diseases (CDs) require comprehensive health care across multiple disciplines. Literature suggests interprofessional collaboration (IPC) in primary care is effective for CD models of care. However, IPC requires innovative implementation, particularly in rural and remote areas where access to health care services and providers is often limited. Our main objective was to identify and synthesize the available research evidence on innovations that promote IPC in primary care for older rural adults with CD, identify gaps in the literature, and provide recommendations for future research.

**Methods:**

Comprehensive and systematic searches were conducted across four scientific databases for peer-reviewed, original research published in English since 1990, resulting in 9,343 records. Following elimination of duplicates, screening, and evaluation, 38 studies were included for synthesis. All studies were described and illustrated by frequency distribution, and findings were grouped thematically.

**Results:**

Most innovations involved case management and focused on diabetes (n = 15), dementia (n = 12), and hypertension (n = 10). Rural challenges were more prevalent than benefits and mainly involved limited services and resources, while strengths were mainly related to close-knit connections and familiarity with one another. Three main themes regarding benefits of the innovations were: 1) enhanced availability/accessibility, 2) earlier detection/management/support, and 3) improved care. Subthemes included: 2a) education/support, 2b) CD or risk factor outcomes, 3a) care continuity, and 3b) care coordination. Five main gaps in the literature included few studies with age-related CDs other than diabetes, dementia, and hypertension; conducted outside of United States and Canada; randomized controlled trial (RCT) and longitudinal studies; that involved virtual or technology-assisted innovations; and that considered sex and gender in the analysis.

**Conclusions:**

Several main areas were highlighted including rural strengths and challenges that impacted the innovations, key innovation benefits, and gaps in the literature. Recommendations for future research were made.

## Introduction

Due to population aging on a global level, between 2020 and 2050 there will be a two-fold increase in the number of older adults aged 60 years and over in the world, to 2.1 billion [1]. Aging is associated with a rise in multi-morbid chronic diseases such as diabetes, chronic cardiovascular and respiratory diseases, cancers, depression, and dementia. Chronic disease, also referred to as noncommunicable disease, requires comprehensive, cross-sector approaches to risk management and innovations at the primary care level that promote early detection and treatment [1,2].

Providing such approaches to care is particularly difficult in rural and remote areas which are typically comprised of proportionally more older adults compared to urban areas, mainly due to declining migration into rural areas [3–5]. Those residing in rural and remote areas typically have limited local access to services and specialists, and experience barriers to accessing services and healthcare providers due to factors such as low socioeconomic status, weak telecommunication infrastructure, and long distance travel to larger centers to access care [6]. The ageing population, geographic isolation, and limited availability of services in rural and remote areas highlight the importance of addressing the healthcare needs of this population [7] in the primary care setting [8–10].

There is a growing body of literature on the effectiveness of interprofessional collaboration (IPC) in primary care chronic disease models of care [8–12]. IPC in healthcare has been described by S. Morgan and colleagues as *“an active and ongoing partnership often between people from diverse backgrounds with distinctive professional cultures and possibly representing different organisations or sectors, who work together to solve problems or provide services”* [8]. An IPC approach involves healthcare providers from at least two different professions/disciplines that work together to provide team-based care (9,10). Examples of primary care professionals include general practitioners (GPs), nurses, paramedics, clinical pharmacists, physiotherapists, physician associates, to name a few [8]. Challenges with implementing an IPC approach in primary care in rural areas have been reported such as limited resources and understanding of roles [13]. Facilitators to implementing the approach in rural areas have also been reported such as smaller team sizes, more frequent team interaction, and more familiarity among team members, with patients, and the community [13].

This scoping review was conducted to identify and synthesize the available research evidence on innovations that promote IPC in primary care for older adults living with chronic disease in rural or remote areas, to identify existing gaps in the literature, and provide recommendations for future research. The three main research questions explored were: what innovations were implemented, what were the rural-related strengths and challenges that impacted those innovations, and what were the benefits of those innovations.

## Methods

The methodological framework of Arksey and O’Malley [14], later built on by Levac, Colquhoun, and O’Brien [15], and the Joanna Briggs Institute [16] was used a priori to develop the protocol for this scoping review a priori. This protocol was not registered.

The five-step framework [14] included: i) identifying the research questions, ii) identifying the relevant studies, iii) study selection, iv) data charting, and v) collating, summarizing, and reporting the results. An iterative, collaborative research team approach was used to develop the research questions, search strategies, and data extraction form. The Preferred Reporting Items for Systematic reviews and Meta-Analyses extension for Scoping Reviews checklist (PRISMA-ScR) [17] was used post-hoc as a rigorous reporting guideline for this review (S1 Appendix).

### Step one: Identifying the research questions

The following three main research questions were explored: 1) What innovations have been implemented in rural and remote areas that involved, supported, promoted, or improved IPC in primary care for older adults living with chronic conditions? 2) What were the strengths and challenges related to rural and remote areas that impacted IPC in primary care for older adults living with chronic conditions? and 3) What were the benefits of providing IPC in primary care to older adults living with chronic conditions in rural and remote communities?

In addition, we explored the following questions: What main themes were identified across the literature? Were any gaps identified in the literature? Were there any differences in findings based on chronic disease(s), individual characteristics (such as participant and provider sex and gender), the role and discipline of the health care professionals involved, or on the basis of study characteristics, such as the research article publication year or country of origin?

### Step two: Identifying the relevant studies

A broad search strategy was designed with the guidance of a university health sciences librarian. Searches were conducted across four scholarly databases (Ovid MEDLINE, Embase, PsycInfo, and Ebsco CINAHL) that included key search terms related to “rural/remote” “primary care” and “interprofessional”. No search terms for chronic disease or older adults were included in the database search strategies as these criteria were manually screened in Step three. Search strategies were customized to each specific database (S2 Appendix). Initial searches were completed on August 30, 2022, and were limited to publications since 1990 and English language only. A subsequent hand-search and forward search were also conducted as described within Step four below.

### Step three: Study selection

All records were first imported to EndNote Desktop Version X8 (Clarivate Analytics, Philadelphia, United States) reference management software and then exported to DistillerSR (Evidence Partners, Ottawa, Canada) systematic review software. After deduplication, remaining items were screened for inclusion. Inclusion and exclusion criteria are described in Table 1. The intent was to include all original, peer-reviewed studies that reported an innovation involving IPC in primary care for older adults living with chronic disease(s) in rural and remote areas. All types of original research methodologies and designs were eligible for inclusion.

**Table 1:** Inclusion criteria.

Peer reviewed, original research (English, 1990 forward)	**The following were excluded**: non-original research, study protocols, reviews, and all grey literature (including, policy papers, reviews, reports, letters to the editor, opinion letters, commentaries, conference abstracts, book chapters, non-peer reviewed documents distributed by both academic and non-academic sources [including web-based resources]).	
**Innovation**	**Innovation** in healthcare could refer to *“a novel idea, product, service or care pathway that has clear benefits when compared to what is currently done. Successful innovations often possess two key qualities: they are both usable and desirable”* [66].	
**Interprofessional, collaboration (IPC) in primary care**	**Interprofessional collaboration:** among two or more healthcare professionals from at least two different professions [9,67] “*who work together to solve problems or provide services*” [and] *“a deeper level of working together in an interdependent way”* [8]. **Primary care:** outside the inpatient setting, and may include nurses, social workers, pharmacists, dietitians, public health practitioners, physicians [68], nurse practitioners, physiotherapists, social workers [69], home care providers, mental healthcare providers, and pharmacists [9].	
**Focus on older adults aged 60 years or more**	If sample is patients, and includes less than 60 years of age, mean age must be 60 + years, or findings for age 60 + must be disaggregated. Older adults defined as age 60+ (as in WHO Fact sheet 2021, updated in 2024 [1]).	
**Focus on age-related chronic condition(s)**	Including but not limited to:	
	• Cancer • Cardiovascular diseases • Chronic kidney disease • Diabetes • High cholesterol • Hypertension	• Mood & Anxiety disorders • Musculoskeletal disorders • Neurological diseases • Oral diseases • Respiratory diseases
	Chronic conditions in older adults operationalized as per Healthy Aging Team 2021, updated in 2024 [70], OECD/European Union 2020 [71], and PHAC 2020 [72].	
**Focus on rural or remote**	If urban data is included, findings must be disaggregated, or majority must be rural.	

**Fig 1:**
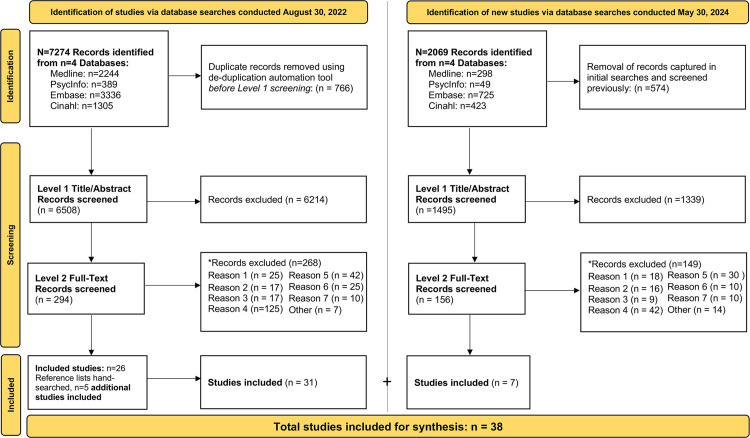
Modified PRISMA flow diagram. *Failure to meet criteria, prioritized in the following order: Reason 1: peer-reviewed original research; 2: solution/innovation; 3: primary care; 4: older adults; 5: rural/remote; 6: interprofessional collaborative primary care approach; 7: age-related chronic disease; 8: other.

Forms were created and piloted in DistillerSR to screen the peer-reviewed literature across two levels. Independent paired authors screened studies for inclusion across both levels of screening. The first author (VE) screened all records, and three coauthors (JK, DPM, CC) each screened one-third of all records at the first level (title/abstract), and at level 2 (full-text) screening. There were no unresolved screening conflicts that required resolution by a fourth coauthor. The search and screen processes are illustrated in the modified PRISMA (Preferred reporting items for systematic reviews and meta-analyses) [18,19] flow diagram (Fig 1).

### Step four: Data charting

The first author extracted key characteristics of the literature including author, year, country, objective/topic, study design and sample, the chronic disease(s), innovation, findings, and author-reported limitations, conclusions, and recommendations relevant to this review. Additional charted data included whether the study was rural/remote or rural-urban, operationalization of ‘rural’ and ‘remote’, the interprofessional primary healthcare professionals involved in the innovation, operationalization of interprofessional collaboration (when provided), and whether there were specialists involved.

Initially data were charted for 26 studies included for synthesis. The reference lists of those studies were then hand-searched, and an additional 5 studies were included for synthesis. A forward search was then conducted on May 30, 2024 (also in S2 Appendix) using the same search strategies and databases outlined in Step 2 above to identify new publications since our initial search conducted August 30, 2022. Records captured in this search (n = 2069) were then cross-referenced with records previously captured and screened in 2022, and 1495 new records underwent similar screening processes outlined in Step 3 above. Data were ultimately charted for an additional n = 7 studies. As reflected in the modified PRISMA (Fig 1), 38 studies were included for synthesis in this review.

### Step five: Collating, summarizing, and reporting the results

The structured approach for this review included both numerical frequency data description and thematic analysis. Studies were collated by publication year, country, methodology, and chronic condition(s), and frequencies of charted study characteristics were selected, counted, and summarized. Thematic analysis [20] of the main findings relevant to the research questions for each of the included studies was conducted using an inductive approach to identify key patterns of meaning in the charted data by reading and re-reading thoroughly and analytically. A semantic approach was used to find common, recurring explicit words or implicit meanings. Data were coded and annotated with color highlighting for emerging themes, and similarities and patterns were named and grouped accordingly.

## Results

As indicated in the modified PRISMA flowchart (Fig 1), 38 original, peer-reviewed studies were included for synthesis [21–58]. Extracted data were collated by publication author, year, country. As shown in a supplementary table (S3 Appendix), the summarized data includes methods, sample, rurality, chronic disease(s), the innovation, primary care providers involved and the nature of their interprofessional collaboration, any specialist involvement in the innovation, rural-related strengths and challenges impacting the innovation, and benefits of the innovation. Most of the data charted in the supplementary table were verbatim extracts from the studies included for synthesis in our review, that may have been paraphrased or summarized for length or clarity.

### Frequencies

Frequency distributions of study characteristics are presented in Table 2.

**Table 2:** Frequencies of study characteristics.

Characteristic	Frequency	Reference numbers
**Country**	--	--
- United States	15	[21,23,25,27,29,31,34,38,40,42,43,45,46,50,52]
- Canada	9	[37,39,41,44,47,51,53,55,56]
- China	4	[22,32,36,54]
- Australia	3	[24,33,49]
- Thailand	3	[28,30,35]
- United Kingdom	2	[57,58]
- Brazil	1	[26]
- India	1	[48]
**Year**	--	--
− 2004–2019	19	[31–43,49,50,55–58]
− 2020–2024	19	[21–30,44–48,51–54]
**Area**	--	--
- Rural	33	[21–23,25,26,28–31,35-58]
- Rural-urban	4	[27,32–34]
- Rural-regional	1	[24]
Definitions of rural (n = 23)	--	[22–24,26–31,33,34,36,38–44,47,53,55,56]
**Study design**		
^+^ RCTs (n = 6), ^*^longitudinal (n = 11)	--	--
- Quantitative	23	[21^*^, 22^ + *^, 23^ + ^, 24–27^*^, 28–31^*^, 32–34^*^, 35^ + *^, 36^ + ^, 37–41^ + *^, 42^ + ^, 43]
- Qualitative	8	[51^*^, 52–55^*^, 56–58]
- Mixed	7	[44^*^, 45–50^*^]
**Study sample**	--	--
- Patients only	23	[22-39, 41–43,48,50]
- Providers only	4	[45,47,53,54]
- Patients, family	4	[46,52,57,58]
- Patients, providers	3	[44,49,56]
- Providers, other health care professionals (HCPs)	2	[51,55]
- Patients, providers, other HCPs	1	[40]
- Patients, providers, family	1	[21]
**Sample sex**	--	--
- Sex reported	30	[22–36,38,39,41–44,46,48,49,53–58]
- More females than males	23	[22,24,26–28,30–32,35,36,38,39,41–44,46,48,49,53–56]
- Sex considered in analysis (sex was not reported in Boise et al. 2020 [40] but was reportedly considered in their analysis)	3	[32,38,40]
**Chronic disease** – categories are not mutually exclusive and may be counted more than once	--	--
- Diabetes ^a^Only diabetes (n = 7)	15	[25^a^,^26^,^27^^a^,^32^,^33^^a^,^34^,^35^,^38^^a^,^39^,^41^,^42^^a^,^43^^a^,^48^,^49^^a^,^50^]
- Dementia ^b^Only dementia (n = 9)	12	[21,24,40,44,45^b^,^47^,^50^^b^,^51^^b^,^52^,^53^,^55^,^58^]
- Hypertension ^c^Only hypertension (n = 2)	10	[22,26,30^c^,^32^^c^,^33^-^36^,^48^,^50^,^54^]
- Depression ^d^Only depression (n = 1)	6	[22,31^d^,^45^,^50^,^51^,^54^]
- Cardiovascular disease ^e^Only cardiovascular disease (n = 1)	5	[23^e^,^32^,^39^,^41^,^50^]
- Chronic obstructive pulmonary disease	3	[39,41,50]
- Chronic kidney disease	2	[28,35]
- Cancer	1	[57]
- Osteoporosis	1	[29]
- Hyperlipidemia	1	[34]
- Nonspecific chronic disease	3	[31,37,46]
**Innovation** - modes of care delivery are not mutually exclusive and may be counted more than once: ^1^virtual (n = 8), ^2^technology-assisted (n = 3), ^3^ in-home (n = 12), in-clinic/other (n = 18)	--	--
- Case management only	18	[22 ^3^ 23 ^2^ 25 ^1^ 27 ^1^ 28 ^3^ 33,34 ^1^ 35 ^3^ 36,38,39 ^3^ 41 ^3^ 42 ^1,3^ 43,46,50 ^1,3^ 54 ^3^, 57]
- Diagnosis and case management	3	[44,53,55]
- Screening and case management	9	[26^2^,^29^,^30^^3^,^32^,^37^^3^,^45^,^48^,^51^^1,3^,^56^]
- Screening, diagnosis, and case management	1	[40]
- Screening and referral	4	[21^1^ 24,31 ^2^ 52]
- Educational	2	[47 ^1^ 49]
- Educational and psychosocial	1	[58^3^]
Studies related to the same innovation that explored different outcomes	--	[21,22,28,35,44,47,52–55]
**Interprofessionals involved in the innovation** – categories are not mutually exclusive and may be counted more than once	--	--
- Physicians	32	[22–24,26–29,31–44,47–50,52–58]
- Nurses (RNs, LPNs, PNs, CNSs)	27	[23–35,37,38,42–44,46–50,53,56,58]
- Pharmacists	18	[23,25,27-30,34,35,38,39,41,43-45,48,50,51,57]
- NPs or Advanced practice nurses	17	[23,25,31,34,37,39–41,44–46,49–51,53,55,56]
- Social workers	11	[21,27–29,44,47,50–53,58]
- Dietitians or nutritionists	11	[25,28,29,35,38,42–45,50,53]
- Occupational or physical therapists	8	[28,35,44,45,47,50,53,55]
- Other clinicians (physician assistants, medical assistants, care aides)	17	[21–23,26,27,31,33,34,36,40,45,46,48–50,52,54]
- Unspecified clinicians	5	[21,25,37,50,52]
- Non-clinicians	20	[23,25,27–31,35,39–41,44,46–48,53,55–58]
- Specialists	18	[21,22,24,25,33,34,37,40,42,44,47,51–55,57,58]

Studies were conducted in eight countries, primarily in North America. Studies included for synthesis were published from 2004 to 2024, with equal numbers published before and after 2020. Most studies were quantitative in design and were comprised of patients only, and included more females than males. Most innovations involved case management, focused on diabetes, dementia, or hypertension, and involved physicians or nurses.

Five main gaps in the literature were identified, including a low number of relevant studies on certain age-related chronic diseases (especially those other than diabetes, dementia, and hypertension), studies conducted in countries outside of the United States and Canada, RCTs and longitudinal studies, studies reporting on virtual and technology-assisted innovations, and studies that considered sex and gender in their analysis.

No differences in the main findings relevant to this scoping review were identified among included studies based on chronic disease, individual or study characteristics, or the health care professionals involved, due to the heterogeneity among studies regarding design, participants, innovations, and outcomes.

### Themes

**Fig 2:**
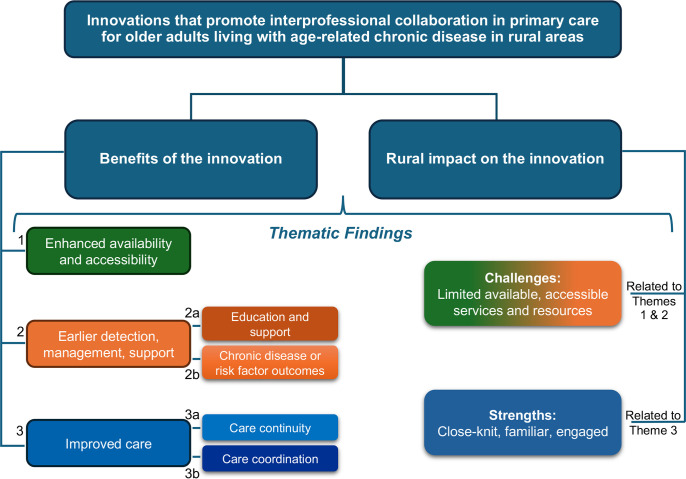
Model of thematic findings.

Thirteen studies [21,26–28,36,37,44,48,50,51,53–55] reported rural-related challenges that impacted their innovation, 3 of which reported strengths and challenges [37,54,55]. Two additional studies also reported related strengths [23,35]. Thematic findings regarding rural challenges were mainly related to limited available, accessible services and resources in general, and are reported below within the context of the three main themes regarding benefits. Rural strengths that impacted the innovations were mainly related to close-knit connections and familiarity with one another, and are presented below, primarily in conjunction with continuity and coordination of care. All 38 studies found benefits of the innovation for patients, reported either directly (by the patients themselves) or indirectly (by providers). Benefits to patients were reported in 36 studies [21–46, 48,50–58], nine that also included benefits for family/caregivers [21,40,44,46,50,52,53,55,58], and for primary care providers (n = 34 studies) [21–27,29,31–42,44–49,51–58]. Thematic analysis of the findings regarding benefits revealed three main themes that reflected the value of the innovations: 1) enhanced availability and accessibility, 2) earlier detection, management, and support, and 3) improved care. Subthemes were also identified for the second and third themes: 2a) education and support, 2b) chronic disease or risk factor outcomes, 3a) care continuity, and 3b) care coordination. Key themes and subthemes are described below and are presented visually as a model in Fig 2, and in Table 3 illustrating themes mapped to corresponding studies.

**Table 3:** Themes mapped to studies.

Studies n = 38	Benefits of the innovation										Rural impact on the innovation	
	to Patients/Caregivers					to Primary Care Providers					Challenges	Strengths
	1	2		3		1	2		3		Related to Themes 1&2. Limited available, accessible services/resources	Related to Theme 3. Close-knit, familiar, engaged
	1	2a	2b*	3a*	3b*	1	2a	2b*	3a*	3b*		
Liu et al. [21]	✔	✔		✔	✔	✔	✔		✔	✔	✔	
Chen et al. [22]			✔					✔				
Partogi et al. [23]	✔			✔	✔	✔	✔		✔	✔		✔
Disler et al. [24]	✔		✔		✔	✔		✔		✔		
Zupa et al. [25]	✔	✔	✔		✔	✔	✔	✔		✔		
Camargo et al. [26]	✔		✔			✔	✔	✔			✔	
Lu et al. [27]	✔		✔	✔	✔	✔	✔	✔	✔	✔	✔	
Thanachayanont et al. [28]	✔	✔	✔		✔			✔		✔	✔	
Wopat et al. [29]	✔		✔		✔	✔	✔	✔		✔		
Woodham et al. [30]	✔		✔	✔	✔			✔	✔	✔		
Burge et al. [31]			✔					✔				
Zheng et al. [32]			✔+				✔	✔+				
Acharya et al. [33]	✔	✔	✔	✔	✔	✔	✔	✔	✔	✔		
Litke et al. [34]	✔		✔	✔	✔	✔		✔	✔	✔		
Jiamjariyapon et al. [35]	✔	✔	✔	✔	✔	✔	✔	✔	✔	✔		✔
Zhang et al. [36]			✔		✔			✔		✔		
Prasad et al. [37]	✔		✔	✔	✔	✔		✔	✔	✔	✔	✔
Bray et al. [38]			✔		✔			✔		✔		
Fletcher et al. [39]			✔					✔				
Boise et al. [40]	✔		✔			✔	✔	✔				
Hogg et al. [41]				✔	✔				✔	✔		
Izquierdo et al. [42]	✔		✔		✔	✔	✔	✔		✔		
Bray et al. [43]			✔					✔				
Morgan et al. [44]	✔	✔			✔	✔	✔			✔	✔	
Kramer et al. [45]	✔				✔	✔	✔			✔		
Cooke et al. [46]	✔		✔	✔	✔	✔		✔	✔	✔		
Kosteniuk et al. [47]					✔	✔	✔			✔		
Lall et al. [48]	✔		✔		✔			✔		✔	✔	
Bonney et al. [49]							✔					
Sorocco et al. [50]	✔		✔	✔				✔	✔		✔	
Schubert et al. [51]	✔			✔	✔	✔			✔	✔	✔	
Bundy et al. [52]	✔			✔		✔			✔			
Morgan et al. [53]	✔	✔	✔	✔	✔	✔	✔	✔	✔	✔	✔	
Li et al. [54]	✔	✔	✔	✔	✔	✔	✔	✔	✔	✔	✔	✔
Morgan et al. [55]	✔		✔	✔	✔	✔	✔	✔	✔	✔	✔	✔
Wong et al. [56]	✔	✔		✔	✔	✔			✔	✔		
Tolson et al. [57]			✔	✔				✔	✔			
Keady et al. [58]	✔	✔	✔	✔		✔	✔	✔	✔			
TOTALS	**27**	**10**	**28**	**19**	**26**	**24**	**19**	**28**	**19**	**26**	**12**	**5**

+greater for rural than urban

*same data for patients/caregivers and primary care providers

1 Enhanced availability and accessibility: Innovations were delivered locally using a mix of formats such as being embedded into routine care, virtual care, and/or in-home care; often filled gaps in services.

2 Earlier detection, management, and support:

2a. Education and support – improved awareness, knowledge, confidence of patients/caregivers and providers; providers learned new skills, standardized tools, and processes.

2b. *Chronic disease or risk factor outcomes – improved patient outcomes and avoided potential health-related crises.

3 Improved care:

3a. *Care continuity – better flexible, patient-centered, whole-person care; consistent, familiar patients and providers were more involved, comfortable, supported.

3b. *Care coordination – better communication, information sharing, collaboration and responsibility sharing among providers.

#### 1) Enhanced availability and accessibility.

Most studies (28/38) reported on innovations that enhanced availability and accessibility of IPC in primary care in terms of healthcare services or other resources [21,23–30,33–35,37,40,42,44–48,50–56,58]. Enhanced availability and accessibility to healthcare services and resources was identified as a key benefit for patients and caregivers (n = 27 studies) [21,23–30,33–35,37,40,42,44–46,48,50–56,58], and for primary care providers (n = 24 studies) [21,23–27,29,33–35,37,40,42,44–47,51–56,58]. Local availability of IPC in primary care services and resources within rural communities resulted in improved access. For example, services were embedded into routine care [23,26,29,30,40,52], delivered via virtual care [21,23,25,27,34,47,51], and via in-home care [28,35,37,54,58]. Studies often reported that innovations filled existing gaps in services [21,23,24,26,27,29,34,42,45,50,53,55,56].

Twelve of those studies [21,26–28,37,44,48,50,51,53–55] also identified rural-related challenges with availability and accessibility of healthcare services and resources that negatively impacted the innovations, including one rural-urban study [27] that reported more challenges for rural clinics than urban. These rural-related challenges mainly centered around limited healthcare services in general [21,27,28,37,44,53], and more specifically in terms of resources such as low numbers of healthcare personnel and technology and/or connectivity. Issues with recruitment, retention and turnover impacted the availability of healthcare personnel to participate in innovations due to staff shortages and high workloads [28,48,53,55]. Travel required to access resulting referrals often posed a distance barrier due to issues such as having no means to travel, and safety concerns related to unpredictable weather or road conditions [26,44,51,54]. Poor or nonexistent internet and cellular connectivity at times impacted innovations, and the technology required to overcome the issues involved using additional equipment and tech-support to connect patients and providers [50,51].

Overall, innovations filled the need for locally available, accessible healthcare services. The next theme follows along that same vein, where having available, accessible healthcare services at the local level often corresponded with earlier detection, management, and support.

#### 2) Earlier detection, management, and support.

Most studies (35/38; with the exception of [39,41,51]), reported on innovations that promoted earlier chronic disease detection, management and support. Innovations could be grouped into two subthemes: education and support (10 studies for patients and/or caregivers [21,25,28,33,35,44,53,54,56,58] and 19 for providers [21,23,25–27,29,32,33,35,40,42,44,45,47,49,53–55,58]), and improvements in chronic disease or risk factor outcomes (28 studies [22,24–40,42,43,46,48,50,53–55,57,58]).

**2a) Education and support.** Innovations in IPC in primary care that involved education and support benefited all (patients, caregivers, and providers) with increased awareness, knowledge, and confidence that improved communication about the chronic disease(s) and facilitated earlier, more frequent referrals to available services and supports. For example, patients and caregivers appreciated being provided with or connected to a variety of chronic disease education and educational resources in general [21,25,28,33,35,44,53,54,56,58], and to additional related services and supports such as other innovations [21,44,56] or organizations [53]. Providers appreciated chronic disease education and training, standardized tools (such as screening tests and technological devices) and processes (such as protocols and best practice guidelines) [21,23,25–27,29,32,33,35,40,42,44,45,47,49,53–55,58], and the opportunity to learn and receive support from each other in the interprofessional collaborative team-based context [21,25,27,29,35,37,38,41,45,47–49,53–56], all of which strengthened providers’ capacity to provide earlier, better care and support for chronic disease management.

**2b) Chronic disease or risk factor outcomes.** Improvements in outcomes were reported across a mix of chronic diseases and risk factors. Improvements included earlier and increased rates of detection and management of chronic disease(s) and risk factors [22,24–40,42,43,46,48,50,53–55,57,58], benefits to general health [32,54] and more specifically in terms of a reduction in inappropriate medication use [39,50], and the reduction of a potential health-related crisis [36,37,42,46,55].

Lastly, in a similar manner, the third and final theme that emerged related to improved care, specifically in terms of better continuity and coordination of care.

#### 3) Improved care.

IPC in primary care is focused on promoting patient care, as reflected in the emergence of the final theme of improved care that centered around two key interrelated concepts, namely care continuity and/or coordination of care in 30/38 studies [21,23–25,27–30,33–38,41,42,44–48,50–58].

**3a) Care continuity.** Related to continuous care over time and trusting relationships between patients/caregivers and providers, improvements to continuity of care were identified in 19 studies [21,23,27,30,33–35,37,41,46,50–58]. Studies with innovations where patients, caregivers, and providers were consistent and familiar to each other [21,27,30,33,37,46,53–56,58], and where efforts were made (such as being flexible and delivering patient-centered care) to build or maintain patient-provider relationships [21,23,27,30,33–35,37,41,51–58], found that these innovations increased comfortability and facilitated communication about needs and supports [21,23,30,33,34,37,46,50–56,58].

**3b) Care coordination.** Improvements in the coordination of care were identified in 26 studies where multiple providers delivered the most appropriate care at the right time [21,23–25,27–30,33–38,41,42,44–48,51,53–56]. Overall, providers reported positive aspects of the IPC in primary care approach regarding coordination of care. Benefits included shared workloads, responsibilities, and support, which promoted interaction and communication, learning from each other, and joint planning and problem-solving [21,23,25,33,35,36,44–46,48,51,53–56]. Providers reported increased awareness of each other’s contributions to care and having more information to work with, which contributed to a more wholistic understanding of the patient, improved capacity to deliver more comprehensive care [21,23–25,27,28,33,36,37,41,42,44–47,51,53–56], and feeling more comfortable with each other and working together [23,33,44,46,53–55]. Several studies found that providers viewed an IPC in primary care approach as a relatively efficient and/or effective approach [21,23,29,30,33,34,36,38,42,44–46,48,53–56].

Rural-related strengths that reportedly had a positive impact on the innovations reflected the close-knit connections in rural communities. For example, rural patients and providers often preferred to have care delivered in their own community where they were already well known to each other [23,37,54,55]. Strengths were mainly centred around the cohesiveness among people including patients and caregivers, providers, and the community in general, with great importance placed on familiarity, comfortability, and engagement with each other and their respective communities [23,35,37,54,55].

## Discussion

Although IPC in primary care has been increasingly identified as the gold standard for comprehensive health care, particularly for older adults living with chronic disease [8–12], innovative ways to deliver such care must be identified for those living in rural areas to effectively overcome geographical barriers such as resource shortages and complexities related to travel [6,7].

This scoping review identified and synthesized 38 peer-reviewed studies on innovations that promoted IPC in primary care for older adults living with age-related chronic disease in rural areas. An increase in the existing literature on this topic was reflected in the equal numbers of identified studies that were published over thirty years, from 1990 to 2020 [31–43,49,50,55–58] and in the subsequent five years, from 2020 to 2024 [21–30,44–48,51–54]. This scoping review identified key benefits attributed to the innovations, and rural-related strengths and challenges that reportedly impacted the innovations. These key areas are discussed in terms of enhanced availability and accessibility; earlier detection, management, and support (including education and support, and chronic disease or risk factor outcomes); and improved care (including care continuity and care coordination).

Overall, innovations that delivered IPC in primary care for older adults living with chronic disease in rural areas improved the availability of, and access to, comprehensive care. We found that the major rural-related challenges reported across multiple studies that negatively impacted the innovations were associated with limited available, accessible services and resources in general. For example, challenges were reflected in shortages in healthcare personnel, travel issues (due to limited means of transportation, and safety concerns with weather or road conditions), and poor or nonexistent internet and cellular connectivity. Despite these challenges, we found that most studies also reported enhanced availability and accessibility overall, for patients and caregivers, and providers. We found a small number of studies reported rural-related strengths that positively impacted the innovations that were mainly centred around the close-knit, cohesive connections of rural people, including patients and caregivers, providers, and the community in general, and the significance of familiarity, comfortability, and the interaction and involvement with each other. Our findings of rural-related strengths and challenges that impacted the innovations in our review are similar to those reported in a scoping review by Perron and colleagues [13] on the limited services and resources in rural areas, and the importance of aspects like familiarity, connection to the community, flexibility, and openness in the rural context.

Innovations largely addressed existing gaps in delivery of interprofessional primary care at the local (rural/remote) level. Rural and remote residents typically experience unique and more access barriers than their urban counterparts, particularly in terms of healthcare provider shortages, necessary travel, and telecommunication infrastructure [6,59]. Often such barriers are even more difficult to navigate for older adults residing in these areas, many of whom experience multi-morbid chronic conditions and require regular access to comprehensive health services from healthcare professionals across a number of disciplines [60]. Given that just two studies included in this review reported virtual or tech-related issues that negatively impacted the innovations, it is perhaps somewhat surprising that few studies (less than a third) utilized virtual or other technology in their innovations. The innovations in the current review delivered IPC in primary care using a variety of formats (such as being embedded into routine care, virtual care, and/or in-home care) that effectively addressed service gaps at the local level, and which fundamentally contributed to enhanced availability and accessibility. A scoping review conducted in 2022 by Montayre et al. [61] on age-friendly innovations in rural and remote areas similarly found that studies included in their review were conducted mainly to address such gaps, often resulting in earlier detection, management, and support.

Patients/caregivers and providers appreciated the opportunity to learn more about chronic diseases and risk factors from the education and support provided in a number of innovations. Education and support improved patient/caregiver ability to communicate and comprehend conversations about their illness, as well as their confidence in managing chronic illness going forward, and more frequent referrals to other services and supports. Similar benefits were among those reported in a review by Stenberg et al. [62] that focused on group-based self-management patient education programs for people with chronic disease, and by Montayre and colleagues [61] where age-friendly innovations with rural and remote older adults were primarily geared toward health promotion programs that improved their ability to age in place. In the current review, providers valued standardized tools, technology, and guidelines, training on their use, and collaborative support and learning from each other in a team-based context, all of which enabled them to deliver earlier, better care and chronic disease management. Similar findings regarding the benefits of shared learning for health professionals were reported in a review by Ojelabi et al. [63] where interprofessional team-based education improved and fostered communication and collaboration among health professionals, in particular for those involved in chronic disease care. Perron and colleagues [13] also identified that the clarification of professional roles and improved teamwork among providers were key outcomes of the studies in their rural review of interprofessional collaboration initiatives in primary care.

In nearly all studies in the current scoping review, innovations facilitated earlier detection of chronic disease, associated risk factors, and other health-related issues. This allowed for more timely support and management, better chronic disease or risk factor outcomes, and fewer health-related crises. We found improved outcomes across a majority of studies across a variety of chronic diseases and risk factors that were associated with earlier, increased rates of detection, management, and support. A small number of these studies reported that innovations were associated with the avoidance of a potential health-related crisis. Similar findings were reported elsewhere, as in a review of systematic reviews by Carron et al. [9] on the effectiveness of using interprofessional collaboration to facilitate consistency in primary care where patient outcomes in general improved in the majority of reviews included in their study. In contrast however, a systematic review conducted by Bouton and colleagues [10] found that although patient outcomes improved across various age groups that were at risk for cardiovascular disease, outcomes for older patients were less clear, as were outcomes for patients with comorbid conditions.

The improvements discussed thus far regarding availability and accessibility, and earlier detection, management, and support, come together as parts of the broader whole, improved care. The construct of improved care associated with the innovations included in this review could be described as care that was not only more accessible and timely, but also more comprehensive, consistent, flexible, and patient-centered, discussed here within the interrelated concepts of continuity and coordination of care.

We found that continuity of care improved in half of all studies included in this review. Studies reported better care continuity for IPC in primary care innovations that involved patients, caregivers, and providers that were familiar with each other, and care was consistently delivered by the same providers. We found that patient-provider relationships were developed and strengthened by innovations that offered flexibility (such as with scheduling and formats) and individualized, person-centered care that added to the comfort level of patients and providers and facilitated discussion about patient needs and supports. These findings are similar to those reported in a scoping review by Perron and colleagues [13] who found in general that familiarity and connection to the community along with flexibility and openness were facilitators of IPC in primary care in the rural context.

We also found improvement in the coordination of care in over half of the studies included in this review. We found that the IPC in primary care approach positively impacted action and communication among providers, and enhanced coordination of care in terms of shared workloads, responsibilities, and support, learning from each other, and joint planning and problem-solving. Sharing information among providers and gaining insight into others’ roles in patient care fostered better understanding of the patient, facilitated delivery of more comprehensive care, and increased comfort with each other and working together collaboratively. Overall, just under half of the studies found that providers perceived the IPC in primary care approach as relatively efficient and/or effective. In general, these findings reflect improved coordination of care and are similar to those reported by Bookey-Bassett and colleagues [64] where better care coordination was reported as a consequence of an IPC in primary care approach for older adults with chronic disease.

### Recommendations and implications

The findings suggest several implications for practice to enhance IPC in rural primary care. Embedding IPC services into local primary care increases accessibility, therefore IPC should be integrated into routine check-ups, virtual consultations, and home visits. Given that poor internet and cellular connectivity may limit IPC innovations, it is essential to ensure adequate technology infrastructure and tech support are available to primary care providers. Educational resources, training, and standardized tools and processes should be provided to primary care providers, as these have been shown to promote earlier detection and management of chronic diseases. Strong patient-provider relationships are key to patient comfort and communication about needs and supports within an IPC context. Delivering patient-centered care through consistent and familiar providers, and supporting mutual understanding and communication among interprofessional teams, may enhance collaboration and more comprehensive patient care.

We found that the existing peer-reviewed literature on IPC in primary care for older adults living with age-related chronic disease in rural and remote areas has increased over time and we identified five main gaps in the literature that could be addressed in future studies. These gaps included a low number of the following: relevant studies on age-related chronic diseases other than diabetes, dementia, and hypertension; studies conducted outside of the United States and Canada; RCTs and longitudinal studies; studies with virtual and technology-assisted innovations; and studies that considered sex and gender in the analysis. More published research is needed in these areas to gain a more thorough understanding of innovations that promote IPC in primary care for older adults living with age-related chronic disease in rural areas. Future studies are needed that concentrate on a wider range of age-related chronic diseases and from countries other than the developed, industrialized nations that made up the bulk of the studies in this review. Rural areas can differ greatly in other parts of the world, especially relative to the United States, where the majority of studies included in this review were conducted and one of the wealthiest nations in the world with no universal health coverage.

Future research should employ longitudinal research designs, and randomized controlled trials, when possible, where long-term follow-up could contribute to a better understanding of changes over time and causal relationships between factors and outcomes. In addition, research on this topic should include a focus on IPC in primary care innovations that use virtual or other technology, represented by less than one third of all studies included in this review. Despite potential challenges to using such technology for health care with older adults in rural areas, innovations have been reported as relatively successful, viable models of care for this population that could be more widely implemented [65]. Lastly, just three studies included in this review considered sex in the analysis. It is possible that the effects of IPC in primary care may differ by sex and/or gender. This is an important area to explore in order to identify and better understand any sex and gender aspects associated with more or less favourable outcomes. Future research should not only report on sex- and gender-based data, but incorporate sex and gender into the analysis and explore potential differences that might inform the development of better personalized services and supports.

Variability among the studies included in this review in terms of design, participants, innovations, and outcomes made comparison difficult and no differences were identified based on chronic disease, individual or study characteristics, or the health care professionals involved.

### Strengths and limitations

A main strength of this scoping review was the systematic, comprehensive, detailed approach to identifying and selecting studies for inclusion. While the search strategy for this review was intentionally broad, it was limited to studies published from 1990 forward, in English only. Therefore, it is possible that some studies may have been missed that could impact the generalizability of our findings. Additionally, most of the literature included for synthesis was from the United States, and comparability was limited due to the heterogeneity of the literature. However, another key strength of this review is that thematic analysis was conducted on the main findings relevant to this review which allowed for a deeper exploration of the data and the identification and interpretation of common patterns and themes. Lastly, publication bias is a potential limitation of scoping reviews because they rely on published literature where less favourable outcomes are less likely to be published. Consistent with other scoping reviews in general, no critical appraisal of the literature was conducted.

## Conclusions

This scoping review synthesized a large number of studies on initiatives that promote IPC in primary care for older adults living with chronic disease in rural and remote areas. We found that although studies were heterogenous and findings were mixed, several areas of importance emerged. We found that innovations most often involved case management, for a number of age-related chronic diseases that most often included diabetes, dementia, and hypertension. The main rural challenge that reportedly impacted the innovations was the limited available, accessible services and resources in general, and the main rural strengths included connections that were close-knit and familiar. Benefits of the innovations were identified and mainly centered around enhanced availability and accessibility of services and resources, contributing to earlier detection, management, and support, and improved coordination and continuity of care. Gaps in the literature were identified and recommendations for future research included: studies on age-related chronic diseases beyond diabetes, dementia, and hypertension; conducting more research outside of the United States and Canada; incorporating randomized controlled trial and longitudinal study designs; exploring virtual or technology-assisted innovations; and integrating sex and gender considerations into analyses.

## Supporting information

S1 Appendix PRISMA-ScR checklist. (DOCX)

S2 Appendix Database searches August 30 2022 and May 30 2024. (DOCX)

S3 Appendix Charted data. (DOCX)

## References

[1] World Health Organization. Ageing and health fact sheet. 2024. Accessed 2025 January 13. https://www.who.int/news-room/fact-sheets/detail/ageing-and-health

[2] World Health Organization. Noncommunicable diseases fact sheet. 2024. Accessed 2025 January 13. https://www.who.int/news-room/fact-sheets/detail/noncommunicable-diseases

[3] United Nations Economic Commission for Europe. Policy brief on ageing No. 18. 2017. https://unece.org/DAM/pau/age/Policy_briefs/ECE-WG1-25-E.pdf

[4] Statistics Canada. Population growth in Canada’s rural areas. Ottawa: Ministry of Industry; 2022. https://www12.statcan.gc.ca/census-recensement/2021/as-sa/98-200-x/2021002/98-200-x2021002-eng.cfm

[5] Davis JC, Cromartie J, Farrigan T, Genetin B, Sanders A, Winikoff JB. Rural America at a glance. United States department of agriculture, economic research service. 2023. 10.32747/2023.8134362.ers

[6] World Health Organization. WHO guideline on health workforce development, attraction, recruitment and retention in rural and remote areas. World Health Organization. 2021. https://iris.who.int/bitstream/handle/10665/341139/9789240024229-eng.pdf 34057827

[7] Hanlon N, Skinner M, Joseph A, Ryser L, Halseth G. New frontiers of rural ageing: resource hinterlands. In: Skinner M, Hanlon N, eds. Ageing resource communities: new frontiers of rural population change, community development and voluntarism. London: Routledge; 2016. 11–23.

[8] Morgan S, Pullon S, McKinlay E. Observation of interprofessional collaborative practice in primary care teams: an integrative literature review. Int J Nurs Stud. 2015;52(7):1217–30. doi: 10.1016/j.ijnurstu.2015.03.008 25862411

[9] Carron T, Rawlinson C, Arditi C, Cohidon C, Hong QN, Pluye P, et al. An Overview of Reviews on Interprofessional Collaboration in Primary Care: Effectiveness. Int J Integr Care. 2021;21(2):31. doi: 10.5334/ijic.5588 34220395 PMC8231476

[10] Bouton C, Journeaux M, Jourdain M, Angibaud M, Huon J-F, Rat C. Interprofessional collaboration in primary care: what effect on patient health? A systematic literature review. BMC Prim Care. 2023;24(1):253. doi: 10.1186/s12875-023-02189-0 38031014 PMC10685527

[11] Kaiser L, Conrad S, Neugebauer EAM, Pietsch B, Pieper D. Interprofessional collaboration and patient-reported outcomes in inpatient care: a systematic review. Syst Rev. 2022;11(1):169. doi: 10.1186/s13643-022-02027-x 35964148 PMC9375378

[12] Fahs I, Akel M, Haddad C, Sacre H, Hajj A, Zeenny RM, et al. Working together for patient health: Assessing interprofessional competencies among healthcare professionals in Lebanon. J Interprof Edu Pract. 2023;32:100630. doi: 10.1016/j.xjep.2023.100630

[13] Perron D, Parent K, Gaboury I, Bergeron DA. Characteristics, barriers and facilitators of initiatives to develop interprofessional collaboration in rural and remote primary healthcare facilities: a scoping review. Rural Remote Health. 2022;22(4):7566. doi: 10.22605/RRH7566 36317229

[14] Arksey H, O’Malley L. Scoping studies: towards a methodological framework. Inter J Soc Res Methodol. 2005;8(1):19–32. doi: 10.1080/1364557032000119616

[15] Levac D, Colquhoun H, O’Brien KK. Scoping studies: advancing the methodology. Implement Sci. 2010;5:69. doi: 10.1186/1748-5908-5-69 20854677 PMC2954944

[16] Peters MDJ, Godfrey C, McInerney P, Munn Z, Tricco AC, Khalil H. Scoping reviews. In: Aromataris E, Lockwood C, Porritt K, Pilla B, Jordan Z, eds. JBI manual for evidence synthesis. JBI; 2024. 417–75.

[17] Tricco AC, Lillie E, Zarin W, O’Brien KK, Colquhoun H, Levac D, et al. PRISMA extension for scoping reviews (PRISMA-ScR): checklist and explanation. Ann Intern Med. 2018;169(7):467–73. doi: 10.7326/M18-0850 30178033

[18] Moher D, Liberati A, Tetzlaff J, Altman DG; PRISMA Group. Preferred reporting items for systematic reviews and meta-analyses: the PRISMA statement. Ann Intern Med. 2009;151(4):264–9, W64. doi: 10.7326/0003-4819-151-4-200908180-00135 19622511

[19] Moher D, Liberati A, Tetzlaff J, Altman DG; PRISMA Group. Preferred reporting items for systematic reviews and meta-analyses: the PRISMA statement. Int J Surg. 2010;8(5):336–41. doi: 10.1016/j.ijsu.2010.02.007 20171303

[20] Braun V, Clarke V. Using thematic analysis in psychology. Qualit Res Psychol. 2006;3(2):77–101. doi: 10.1191/1478088706qp063oa

[21] Liu T-L, Woodward JM, Frazier L, Rossman W, Taylor YJ, Mangieri DA. Transitioning an in-person geriatric memory clinic to a virtual care model for rural primary care clinics. J Am Geriatr Soc. 2022;70(7):2156–61. doi: 10.1111/jgs.17772 35398891

[22] Chen S, Conwell Y, Xue J, Li L, Zhao T, Tang W, et al. Effectiveness of integrated care for older adults with depression and hypertension in rural China: a cluster randomized controlled trial. PLoS Med. 2022;19(10):e1004019. doi: 10.1371/journal.pmed.1004019 36279299 PMC9639850

[23] Partogi M, Gaviria-Valencia S, Alzate Aguirre M, Pick NJ, Bhopalwala HM, Barry BA, et al. Sociotechnical intervention for improved delivery of preventive cardiovascular care to rural communities: participatory design approach. J Med Internet Res. 2022;24(8):e27333. doi: 10.2196/27333 35994324 PMC9446142

[24] Disler R, Pascoe A, Anderson H, Piejko E, Asaid A, Disler P. A new model for general practice-led, regional, community-based, memory clinics. BMC Prim Care. 2022;23(1):242. doi: 10.1186/s12875-022-01829-1 36127660 PMC9487024

[25] Zupa MF, Beattie J, Boudreaux-Kelly M, Larson M, Lumley B, Lutz-McCain S, et al. Diabetes care network: a novel model to disseminate team-based diabetes specialty care in a rural population. Sci Diabetes Self Manag Care. 2022;48(6):483–91. doi: 10.1177/26350106221125690 36125114 PMC9691529

[26] Camargo MS, Passos LCS, Mistro S, Soares DA, Kochergin CN, de Carvalho VCHDS, et al. Improving access to the glycated hemoglobin test in rural communities with point-of-care devices: an application study. Front Med (Lausanne). 2021;8:734306. doi: 10.3389/fmed.2021.734306 34881257 PMC8645789

[27] Lu A, Gunzburger E, Glorioso T, Smith W, Whooley M, Ho M. Impact of longitudinal virtual primary care on diabetes quality of care. Health Serv Res. 2020;55(S1):81–2. doi: 10.1111/1475-6773.13443 PMC7822396 33483815

[28] Thanachayanont T, Chanpitakkul M, Hengtrakulvenit J, Watcharakanon P, Wisansak W, Tancharoensukjit T, et al. Effectiveness of integrated care on delaying chronic kidney disease progression in rural communities of Thailand (ESCORT-2) trials. Nephrology (Carlton). 2021;26(4):333–40. doi: 10.1111/nep.13849 33442912 PMC7986192

[29] Wopat M, Breslow R, Chesney K, McCauley M, Van Gysel R, Gray A, et al. Implementation of a pharmacist and student pharmacist-led primary care service to identify and treat rural veterans at risk for osteoporotic fracture. J Am Pharm Assoc (2003). 2021;61(6):e105–12. doi: 10.1016/j.japh.2021.07.011 34393078

[30] Woodham NS, Taneepanichskul S, Somrongthong R, Kitsanapun A, Sompakdee B. Effectiveness of a multidisciplinary approach intervention to improve blood pressure control among elderly hypertensive patients in rural thailand: a quasi-experimental study. J Multidiscip Healthc. 2020;13:571–80. doi: 10.2147/JMDH.S254286 32694916 PMC7340360

[31] Burge SA, Powell W, Mazour L. A quality improvement endeavor improving depression screening for rural older adults. Online J Rural Nurs Health Care. 2019;19(2):44–64. doi: 10.14574/ojrnhc.v19i2.563

[32] Zheng X, Xiao F, Li R, Yin D, Xin Q, Yang H, et al. The effectiveness of hypertension management in China: a community-based intervention study. Prim Health Care Res Dev. 2019;20:e111. doi: 10.1017/S1463423618000853 32799973 PMC6635806

[33] Acharya S, Philcox AN, Parsons M, Suthers B, Luu J, Lynch M, et al. Hunter and New England diabetes alliance: innovative and integrated diabetes care delivery in general practice. Aust J Prim Health. 2019;25:219–43. doi: 10.1071/PY18179 31221243

[34] Litke J, Spoutz L, Ahlstrom D, Perdew C, Llamas W, Erickson K. Impact of the clinical pharmacy specialist in telehealth primary care. Am J Health Syst Pharm. 2018;75(13):982–6. doi: 10.2146/ajhp170633 29941537

[35] Jiamjariyapon T, Ingsathit A, Pongpirul K, Vipattawat K, Kanchanakorn S, Saetie A, et al. Effectiveness of integrated care on delaying progression of stage 3-4 chronic kidney disease in rural communities of Thailand (ESCORT study): a cluster randomized controlled trial. BMC Nephrol. 2017;18(1):83. doi: 10.1186/s12882-016-0414-4 28253839 PMC5335731

[36] Zhang Y, Tang W, Zhang Y, Liu L, Zhang L. Effects of integrated chronic care models on hypertension outcomes and spending: a multi-town clustered randomized trial in China. BMC Public Health. 2017;17(1):244. doi: 10.1186/s12889-017-4141-y 28284202 PMC5346199

[37] Prasad S, Dunn W, Hillier LM, McAiney CA, Warren R, Rutherford P. Rural geriatric glue: a nurse practitioner–led model of care for enhancing primary care for frail older adults within an ecosystem approach. J American Geriatrics Society. 2014;62(9):1772–80. doi: 10.1111/jgs.12982 25243682

[38] Bray P, Cummings DM, Morrissey S, Thompson D, Holbert D, Wilson K, et al. Improved outcomes in diabetes care for rural African Americans. Ann Fam Med. 2013;11(2):145–50. doi: 10.1370/afm.1470 23508601 PMC3601402

[39] Fletcher J, Hogg W, Farrell B, Woodend K, Dahrouge S, Lemelin J, et al. Effect of nurse practitioner and pharmacist counseling on inappropriate medication use in family practice. Canadian Family Physician. 2012;58(8):862–8. 22893340 PMC3418988

[40] Boise L, Eckstrom E, Fagnan L, King A, Goubaud M, Buckley DI, et al. The rural older adult memory (ROAM) study: a practice-based intervention to improve dementia screening and diagnosis. J Am Board Fam Med. 2010;23(4):486–98. doi: 10.3122/jabfm.2010.04.090225 20616291 PMC3627347

[41] Hogg W, Lemelin J, Dahrouge S, Liddy C, Armstrong CD, Legault F, et al. Randomized controlled trial of anticipatory and preventive multidisciplinary team care: for complex patients in a community-based primary care setting. Canadian Family Physician. 2009;55(12):e76-85. 20008582 PMC2793206

[42] Izquierdo R, Meyer S, Starren J, Goland R, Teresi J, Shea S, et al. Detection and remediation of medically urgent situations using telemedicine case management for older patients with diabetes mellitus. Ther Clin Risk Manag. 2007;3(3):485–9. 18488079 PMC2386361

[43] Bray P, Thompson D, Wynn JD, Cummings DM, Whetstone L. Confronting disparities in diabetes care: the clinical effectiveness of redesigning care management for minority patients in rural primary care practices. J Rural Health. 2005;21(4):317–21. doi: 10.1111/j.1748-0361.2005.tb00101.x 16294654

[44] Morgan DG, Kosteniuk J, Bayly M. Perceptions and outcomes of an embedded Alzheimer society first link coordinator in rural primary health care memory clinics. BMC Health Serv Res. 2024;24(1):607. doi: 10.1186/s12913-024-11066-0 38724975 PMC11080231

[45] Kramer BJ, Weintraub NT, Richter-Lagha RA. Infusing geriatrics in Indian Health Service general primary care clinics: extending VA workforce development training. Gerontol Geriatr Educ. 2023;44(3):354–63. doi: 10.1080/02701960.2022.2056735 35377832

[46] Cook KL, Mayahara M, Tivis L. Evaluation of the nurse practitioner offsite model. J Gerontol Nurs. 2023;49(7):25–30. doi: 10.3928/00989134-20230615-05 37379050

[47] Kosteniuk J, Morgan D, O’Connell ME, Seitz D, Elliot V, Bayly M, et al. Dementia-related continuing education for rural interprofessional primary health care in Saskatchewan, Canada: perceptions and needs of webinar participants. Prim Health Care Res Dev. 2022;23:e32. doi: 10.1017/S1463423622000226 35604026 PMC9247685

[48] Lall D, Engel N, Srinivasan PN, Devadasan N, Horstman K, Criel B. Improving primary care for diabetes and hypertension: findings from implementation research in rural South India. BMJ Open. 2020;10(12):e040271. doi: 10.1136/bmjopen-2020-040271 33323433 PMC7745330

[49] Bonney A, Dijkmans-Hadley B, Seidel B, MacKinnon D, Phillipson L. A feasibility study of team-based primary care for chronic disease management training in rural Australia. Aust J Rural Health. 2017;25(1):66–7. doi: 10.1111/ajr.12289 27087105

[50] Sorocco KH, Bratkovich KL, Wingo R, Qureshi SM, Mason PJ. Integrating care coordination home telehealth and home based primary care in rural Oklahoma: a pilot study. Psychol Serv. 2013;10(3):350–2. doi: 10.1037/a0032785 23937085

[51] Schubert CC, Penney LS, Schwartzkopf AL, Damush TM, Preddie A, Flemming S, et al. Expanding access to comprehensive geriatric evaluation via telehealth: development of hybrid-virtual home visits. J Gen Intern Med. 2024;39(Suppl 1):36–43. doi: 10.1007/s11606-023-08460-5 38227169 PMC10937878

[52] Bundy H, Frazier L, Woodward JM, Liu T-L, Taylor YJ, Rossman W, et al. The benefits of virtual in-clinic memory care for rural patients with dementia: preliminary data. J Am Geriatr Soc. 2022;70(6):1874–6. doi: 10.1111/jgs.17712 35211952

[53] Morgan D, Kosteniuk J, O’Connell ME, Seitz D, Elliot V, Bayly M, et al. Factors influencing sustainability and scale-up of rural primary healthcare memory clinics: perspectives of clinic team members. BMC Health Serv Res. 2022;22(1):148. doi: 10.1186/s12913-022-07550-0 35120516 PMC8814777

[54] Li LW, Xue J, Conwell Y, Yang Q, Chen S. Implementing collaborative care for older people with comorbid hypertension and depression in rural China. Int Psychogeriatr. 2020;32(12):1457–65. doi: 10.1017/S1041610219001509 31630703 PMC7170762

[55] Morgan D, Kosteniuk J, O’Connell ME, Kirk A, Stewart NJ, Seitz D, et al. Barriers and facilitators to development and implementation of a rural primary health care intervention for dementia: a process evaluation. BMC Health Serv Res. 2019;19(1):709. doi: 10.1186/s12913-019-4548-5 31623609 PMC6798332

[56] Wong S, Browne A, Lavoie J, Macleod M, Chongo M, Ulrich C. Incorporating group medical visits into primary healthcare: are there benefits?. hcpol. 2015;11(2):27–42. doi: 10.12927/hcpol.2016.24449 PMC4729281 26742114

[57] Tolson D, McIntosh J, Loftus L, Cormie P. Developing a managed clinical network in palliative care: a realistic evaluation. Int J Nurs Stud. 2007;44(2):183–95. doi: 10.1016/j.ijnurstu.2005.11.027 16423354

[58] Keady J, Woods B, Hahn S, Hill J. Community mental health nursing and early intervention in dementia: developing practice through a single case history. J Clin Nurs. 2004;13(6B):57–67. doi: 10.1111/j.1365-2702.2004.01045.x 15724820

[59] Cohen SA, Greaney ML. Aging in rural communities. Curr Epidemiol Rep. 2023;10(1):1–16. doi: 10.1007/s40471-022-00313-9 36404874 PMC9644394

[60] Golembiewski EH, Gravholt DL, Torres Roldan VD, Lincango Naranjo EP, Vallejo S, Bautista AG, et al. Rural patient experiences of accessing care for chronic conditions: a systematic review and thematic synthesis of qualitative studies. Ann Fam Med. 2022;20(3):266–72. doi: 10.1370/afm.2798 35606138 PMC9199043

[61] Montayre J, Foster J, Zhao IY, Kong A, Leung AYM, Molassiotis A, et al. Age-friendly interventions in rural and remote areas: a scoping review. Australas J Ageing. 2022;41(4):490–500. doi: 10.1111/ajag.13101 35796240 PMC10083949

[62] Stenberg U, Haaland-Øverby M, Fredriksen K, Westermann KF, Kvisvik T. A scoping review of the literature on benefits and challenges of participating in patient education programs aimed at promoting self-management for people living with chronic illness. Patient Educ Counsel. 2016;99(11):1759–71. doi: 10.1016/j.pec.2016.07.027 27461944

[63] Ojelabi AO, Ling J, Roberts D, Hawkins C. Does interprofessional education support integration of care services? A systematic review. J Interprof Edu Pract. 2022;28:100534. doi: 10.1016/j.xjep.2022.100534

[64] Bookey‐Bassett S, Markle‐Reid M, Mckey CA, Akhtar‐Danesh N. Understanding interprofessional collaboration in the context of chronic disease management for older adults living in communities: a concept analysis. J Adv Nurs. 2016;73(1):71–84. doi: 10.1111/jan.13162 27681818

[65] Savira F, Gupta A, Gilbert C, Huggins CE, Browning C, Chapman W, et al. Virtual care initiatives for older adults in Australia: scoping review. J Med Internet Res. 2023;25:e38081. doi: 10.2196/38081 PMC9892987 36652291

[66] Kelly CJ, Young AJ. Promoting innovation in healthcare. Future Healthc J. 2017;4(2):121–5. doi: 10.7861/futurehosp.4-2-121 31098448 PMC6502619

[67] Gilbert JHV, Yan J, Hoffman SJ. A WHO report: framework for action on interprofessional education and collaborative practice. J Allied Health. 2010;39 Suppl 1:196–7. 21174039

[68] Canadian Institutes of Health Research CIHR. Community-based primary health care overview. 2015. https://cihr-irsc.gc.ca/e/44079.html

[69] Canadian Institute for Health Information CIHI. Primary care. 2023. Accessed 2025 May 10. https://www.cihi.ca/en/topics/primary-care

[70] Healthy Aging Team. Chronic conditions in older adults. 2024. Accessed 2024 October 20. https://www.ncoa.org/article/the-top-10-most-common-chronic-conditions-in-older-adults

[71] OECD/European Union. Chronic diseases and disabilities among older people. Health at a glance: Europe 2020: state of health in the EU cycle. Paris, France: OECD Publishing; 2020. 132–4.

[72] Government of Canada. Aging and chronic diseases: a profile of Canadian seniors. HP35-137/1-2020E-PDF. Public Health Agency of Canada. 2020. https://www.canada.ca/content/dam/hc-sc/documents/services/publications/diseases-and-conditions/aging-chronic-diseases/canadian-seniors-report_2021-eng.pdf

