# Supplementary material for: A scoping review of innovations that promote interprofessional collaboration (IPC) in primary care for older adults living with age-related chronic disease in rural areas
Source: PLoS One. 2025 Sep 3;20(9):e0331327. doi: 10.1371/journal.pone.0331327 (PMC12407450; doi:10.1371/journal.pone.0331327)
Supplement: S2 Appendix — (DOCX) [file pone.0331327.s002.docx]

**S2 Appendix. Database searches August 30, 2022 and May 30, 2024**

**August 30, 2022**

**Ovid Embase 1988 to 2022 (English, 1990=>); n=3336**

| 1 | (rural* or remote* or isolat* or north* or "sparse population" or "sparse populations").mp. | 2555548 |
| --- | --- | --- |
| 2 | rural health/ or urban rural difference/ or rural area/ or rural population/ or exp rural health care/ | 124905 |
| 3 | 1 or 2 | 2555548 |
| 4 | (primary adj3 team*).tw. | 5896 |
| 5 | primary health care/ or primary medical care/ | 187653 |
| 6 | ambulatory care/ or ambulatory care nursing/ or ambulatory monitoring/ | 46463 |
| 7 | (outpatient adj2 (care or service*)).tw. | 23586 |
| 8 | general practice/ | 72132 |
| 9 | group practice/ | 6196 |
| 10 | (primary adj2 care).tw. | 203982 |
| 11 | (primary adj2 healthcare).tw. | 9967 |
| 12 | 4 or 5 or 6 or 7 or 8 or 9 or 10 or 11 | 383288 |
| 13 | team nursing/ | 442 |
| 14 | (inter-professional* or interprofessional*).tw. | 18460 |
| 15 | (inter-professional* or interprofessional*).tw. | 18460 |
| 16 | (inter-occupation* or interoccupation*).tw. | 12 |
| 17 | (multi-profession* or multiprofession*).tw. | 5101 |
| 18 | (multi-disciplin* or multidisciplin*).tw. | 185072 |
| 19 | (multi-occupation* or multioccupation*).tw. | 43 |
| 20 | (trans-discipinar* or transdisciplinar*).tw. | 2893 |
| 21 | (cross-occupation or crossoccupation).tw. | 2 |
| 22 | (cross-disciplin* or crossdisciplin*).tw. | 2698 |
| 23 | (cross-profession* or crossprofession*).tw. | 168 |
| 24 | interdisciplinary communication/ | 13064 |
| 25 | team*.mp. | 334571 |
| 26 | doctor nurse relation/ | 7567 |
| 27 | 13 or 14 or 15 or 16 or 17 or 18 or 19 or 20 or 21 or 22 or 23 or 24 or 25 or 26 | 480138 |
| 28 | 3 and 12 and 27 | 3481 |
| 29 | limit 28 to (english language and yr="1990 -Current") | **3336** |

**Ovid APA PsycInfo 1987 to August Week 4 2022 (English, 1990=>); n=389**

| 1 | (rural* or remote* or isolat* or north* or "sparse population" or "sparse populations").mp. | 178579 |
| --- | --- | --- |
| 2 | exp RURAL ENVIRONMENTS/ | 18275 |
| 3 | 1 or 2 | 178579 |
| 4 | (primary adj3 team*).tw. | 1049 |
| 5 | primary health care/ | 20264 |
| 6 | outpatient treatment/ or outpatient commitment/ | 6398 |
| 7 | (outpatient adj2 (care or service*)).tw. | 4777 |
| 8 | ((general or family) adj practic*).tw. | 7480 |
| 9 | (primary adj2 healthcare).tw. | 1505 |
| 10 | or/4-9 | 36991 |
| 11 | exp Interdisciplinary Treatment Approach/ | 6676 |
| 12 | (inter-professional* or interprofessional*).tw. | 5453 |
| 13 | (inter-disciplin* or interdisciplin*).tw. | 25589 |
| 14 | (inter-occupation* or interoccupation*).tw. | 18 |
| 15 | (multi-profession* or multiprofession*).tw. | 989 |
| 16 | (multi-disciplin* or multidisciplin*).tw. | 22755 |
| 17 | (multi-occupation* or multioccupation*).tw. | 43 |
| 18 | (trans-discipinar* or transdisciplinar*).tw. | 1996 |
| 19 | (cross-occupation or crossoccupation).tw. | 7 |
| 20 | (cross-disciplin* or crossdisciplin*).tw. | 2198 |
| 21 | (cross-profession* or crossprofession*).tw. | 76 |
| 22 | team*.mp. | 90289 |
| 23 | integrated services/ | 4772 |
| 24 | 11 or 12 or 13 or 14 or 15 or 16 or 17 or 18 or 19 or 20 or 21 or 22 or 23 | 137568 |
| 25 | 3 and 10 and 24 | 410 |
| 26 | limit 25 to (english language and yr="1990 -Current") | **389** |

**Ovid MEDLINE(R) 1946 to August Week 4 2022 (English, 1990=>); n=2244**

| 1 | RURAL HEALTH/ or RURAL POPULATION/ or HOSPITALS, RURAL/ or RURAL NURSING/ or RURAL HEALTH SERVICES/ | 104000 |
| --- | --- | --- |
| 2 | (rural* or remote* or isolat* or north* or "sparse population" or "sparse populations").mp. | 2576580 |
| 3 | 1 or 2 | 2576580 |
| 4 | (primary adj3 team*).tw. | 3511 |
| 5 | Primary Health Care/ | 88857 |
| 6 | Ambulatory Care/ | 45839 |
| 7 | (outpatient adj2 (care or service*)).tw. | 13995 |
| 8 | general practice/ or family practice/ | 77701 |
| 9 | Group Practice/ | 7922 |
| 10 | (primary adj2 care).tw. | 138124 |
| 11 | (primary adj2 healthcare).tw. | 6922 |
| 12 | or/4-11 | 286860 |
| 13 | patient care team/ or nursing, team/ | 71333 |
| 14 | INTERPROFESSIONAL RELATIONS/ | 52935 |
| 15 | (inter-professional* or interprofessional*).tw. | 11956 |
| 16 | (inter-disciplin* or interdisciplin*).tw. | 37819 |
| 17 | (inter-occupation* or interoccupation*).tw. | 11 |
| 18 | (multi-profession* or multiprofession*).tw. | 2700 |
| 19 | (multi-disciplin* or multidisciplin*).tw. | 94317 |
| 20 | (multi-occupation* or multioccupation*).tw. | 29 |
| 21 | (trans-discipinar* or transdisciplinar*).tw. | 1931 |
| 22 | (cross-occupation or crossoccupation).tw. | 1 |
| 23 | (cross-disciplin* or crossdisciplin*).tw. | 1777 |
| 24 | (cross-profession* or crossprofession*).tw. | 124 |
| 25 | Interdisciplinary Communication/ | 18099 |
| 26 | team*.mp. | 218971 |
| 27 | physician-nurse relations/ | 2401 |
| 28 | 13 or 14 or 15 or 16 or 17 or 18 or 19 or 20 or 21 or 22 or 23 or 24 or 25 or 26 or 27 | 363007 |
| 29 | 3 and 12 and 28 | 2620 |
| 30 | limit 29 to (english language and yr="1990 -Current") | **2244** |

**Ebsco – CINAHL (English, 1990=>, excluding MEDLINE); n=1305**

| S1 | TI (primary N3 team*) OR AB (primary N3 team*) | Search modes - Boolean/Phrase |  |
| --- | --- | --- | --- |
| S2 | (MH "Multidisciplinary Care Team") | Search modes - Boolean/Phrase |  |
| S3 | (MH "Team Nursing") | Search modes - Boolean/Phrase |  |
| S4 | (MH "Primary Health Care") | Search modes - Boolean/Phrase |  |
| S5 | (MH "Ambulatory Care") | Search modes - Boolean/Phrase |  |
| S6 | TI ( (outpatient N2 (care or service*)) ) OR AB ( (outpatient N2 (care or service*)) ) | Search modes - Boolean/Phrase |  |
| S7 | (MH "Family Practice") | Search modes - Boolean/Phrase |  |
| S8 | (MH "Group Practice") OR (MH "Joint Practice") | Search modes - Boolean/Phrase |  |
| S9 | TI (primary N2 care) OR AB (primary N2 care) | Search modes - Boolean/Phrase |  |
| S10 | TI (primary N2 healthcare) OR AB (primary N2 healthcare) | Search modes - Boolean/Phrase |  |
| S11 | S1 OR S2 OR S3 OR S4 OR S5 OR S6 OR S7 OR S8 OR S9 OR S10 | Search modes - Boolean/Phrase |  |
| S12 | (MH "Rural Health Personnel") OR (MH "Rural Health Centers") OR (MH "Hospitals, Rural") OR (MH "Rural Population") OR (MH "Rural Health Services") OR (MH "Australian Rural Nurses and Midwives") OR (MH "Rural Health Nursing") OR (MH "Rural Areas") OR (MH "Association for Australian Rural Nurses") OR (MH "Services for Australian Rural and Remote Allied Health") OR (MH "Rural Health") OR (MH "Frontier Nursing Service") | Search modes - Boolean/Phrase |  |
| S13 | (rural* or remote* or isolat* or north* or "sparse population" or "sparse populations") | Search modes - Boolean/Phrase |  |
| S14 | S12 OR S13 | Search modes - Boolean/Phrase |  |
| S15 | (MH "Interprofessional Relations") | Search modes - Boolean/Phrase |  |
| S16 | TI ( (inter-professional* or interprofessional*) ) OR AB ( (inter-professional* or interprofessional*) ) | Search modes - Boolean/Phrase |  |
| S17 | TI ( (inter-disciplin* or interdisciplin*) ) OR AB ( (inter-disciplin* or interdisciplin*) ) | Search modes - Boolean/Phrase |  |
| S18 | TI ( (inter-occupation* or interoccupation*) ) OR AB ( (inter-occupation* or interoccupation*) ) | Search modes - Boolean/Phrase |  |
| S19 | TI ( (multi-profession* or multiprofession*) ) OR AB ( (multi-profession* or multiprofession*) ) | Search modes - Boolean/Phrase |  |
| S20 | TI ( (multi-disciplin* or multidisciplin*) ) OR AB ( (multi-disciplin* or multidisciplin*) ) | Search modes - Boolean/Phrase |  |
| S21 | TI ( (multi-occupation* or multioccupation*) ) OR AB ( (multi-occupation* or multioccupation*) ) | Search modes - Boolean/Phrase |  |
| S22 | TI ( (trans-discipinar* or transdisciplinar*) ) OR AB ( (trans-discipinar* or transdisciplinar*) ) | Search modes - Boolean/Phrase |  |
| S23 | TI ( (cross-occupation or crossoccupation) ) OR AB ( (cross-occupation or crossoccupation) ) | Search modes - Boolean/Phrase |  |
| S24 | TI ( (cross-disciplin* or crossdisciplin*) ) OR AB ( (cross-disciplin* or crossdisciplin*) ) | Search modes - Boolean/Phrase |  |
| S25 | TI ( (cross-profession* or crossprofession*) ) OR AB ( (cross-profession* or crossprofession*) ) | Search modes - Boolean/Phrase |  |
| S26 | TI team* OR AB team* | Search modes - Boolean/Phrase |  |
| S27 | (MH "Nurse-Physician Relations") | Search modes - Boolean/Phrase |  |
| S28 | S15 OR S16 OR S17 OR S18 OR S19 OR S20 OR S21 OR S22 OR S23 OR S24 OR S25 OR S26 OR S27 | Search modes - Boolean/Phrase |  |
| S29 | S11 AND S14 AND S28 | Expanders - Apply equivalent subjects  Search modes - Boolean/Phrase | Results (2,812) |
| S30 | S11 AND S14 AND S28 | Limiters - Published Date: 19900101-20220931; English Language; Peer Reviewed; Exclude MEDLINE records  Expanders - Apply equivalent subjects  Search modes - Boolean/Phrase | Results (**1,305**) |

**May 30, 2024**

**Ovid Embase 1988 to 2024 (English, 2022=>current); n=997**

| 1 | (rural* or remote* or isolat* or north* or "sparse population" or "sparse populations").mp. | 2832131 |
| --- | --- | --- |
| 2 | rural health/ or urban rural difference/ or rural area/ or rural population/ or exp rural health care/ | 141132 |
| 3 | 1 or 2 | 2832131 |
| 4 | (primary adj3 team*).tw. | 6874 |
| 5 | primary health care/ or primary medical care/ | 210102 |
| 6 | ambulatory care/ or ambulatory care nursing/ or ambulatory monitoring/ | 49414 |
| 7 | (outpatient adj2 (care or service*)).tw. | 27356 |
| 8 | general practice/ | 76673 |
| 9 | group practice/ | 6386 |
| 10 | (primary adj2 care).tw. | 229810 |
| 11 | (primary adj2 healthcare).tw. | 12319 |
| 12 | 4 or 5 or 6 or 7 or 8 or 9 or 10 or 11 | 424474 |
| 13 | team nursing/ | 510 |
| 14 | (inter-professional* or interprofessional*).tw. | 22083 |
| 15 | (inter-professional* or interprofessional*).tw. | 22083 |
| 16 | (inter-occupation* or interoccupation*).tw. | 12 |
| 17 | (multi-profession* or multiprofession*).tw. | 5930 |
| 18 | (multi-disciplin* or multidisciplin*).tw. | 221535 |
| 19 | (multi-occupation* or multioccupation*).tw. | 45 |
| 20 | (trans-discipinar* or transdisciplinar*).tw. | 3505 |
| 21 | (cross-occupation or crossoccupation).tw. | 2 |
| 22 | (cross-disciplin* or crossdisciplin*).tw. | 3210 |
| 23 | (cross-profession* or crossprofession*).tw. | 185 |
| 24 | interdisciplinary communication/ | 13695 |
| 25 | team*.mp. | 406142 |
| 26 | doctor nurse relation/ | 8517 |
| 27 | 13 or 14 or 15 or 16 or 17 or 18 or 19 or 20 or 21 or 22 or 23 or 24 or 25 or 26 | 576075 |
| 28 | 3 and 12 and 27 | 4303 |
| 29 | limit 28 to (english language and yr="2022 -Current") | **997** |

**Ovid APA PsycInfo <1987 to May Week 4 2024> (English, 2022=>current); n=68**

| 1 | (rural* or remote* or isolat* or north* or "sparse population" or "sparse populations").mp. | 199418 |
| --- | --- | --- |
| 2 | exp RURAL ENVIRONMENTS/ | 21310 |
| 3 | 1 or 2 | 199418 |
| 4 | (primary adj3 team*).tw. | 1176 |
| 5 | primary health care/ | 22395 |
| 6 | outpatient treatment/ or outpatient commitment/ | 7242 |
| 7 | (outpatient adj2 (care or service*)).tw. | 5333 |
| 8 | ((general or family) adj practic*).tw. | 7896 |
| 9 | (primary adj2 healthcare).tw. | 1791 |
| 10 | or/4-9 | 40793 |
| 11 | exp Interdisciplinary Treatment Approach/ | 7059 |
| 12 | (inter-professional* or interprofessional*).tw. | 6387 |
| 13 | (inter-disciplin* or interdisciplin*).tw. | 28261 |
| 14 | (inter-occupation* or interoccupation*).tw. | 18 |
| 15 | (multi-profession* or multiprofession*).tw. | 1133 |
| 16 | (multi-disciplin* or multidisciplin*).tw. | 25371 |
| 17 | (multi-occupation* or multioccupation*).tw. | 51 |
| 18 | (trans-discipinar* or transdisciplinar*).tw. | 2348 |
| 19 | (cross-occupation or crossoccupation).tw. | 8 |
| 20 | (cross-disciplin* or crossdisciplin*).tw. | 2426 |
| 21 | (cross-profession* or crossprofession*).tw. | 83 |
| 22 | team*.mp. | 100535 |
| 23 | integrated services/ | 5784 |
| 24 | 11 or 12 or 13 or 14 or 15 or 16 or 17 or 18 or 19 or 20 or 21 or 22 or 23 | 153249 |
| 25 | 3 and 10 and 24 | 495 |
| 26 | limit 25 to (english language and yr="2022 -Current") | **68** |

**Ovid MEDLINE(R) 1946 to May Week 4 2024 (English, 2022=>current); n=427**

| 1 | RURAL HEALTH/ or RURAL POPULATION/ or HOSPITALS, RURAL/ or RURAL NURSING/ or RURAL HEALTH SERVICES/ | 107405 |
| --- | --- | --- |
| 2 | (rural* or remote* or isolat* or north* or "sparse population" or "sparse populations").mp. | 2694325 |
| 3 | 1 or 2 | 2694325 |
| 4 | (primary adj3 team*).tw. | 3949 |
| 5 | Primary Health Care/ | 94198 |
| 6 | Ambulatory Care/ | 46843 |
| 7 | (outpatient adj2 (care or service*)).tw. | 15703 |
| 8 | general practice/ or family practice/ | 79355 |
| 9 | Group Practice/ | 7960 |
| 10 | (primary adj2 care).tw. | 151673 |
| 11 | (primary adj2 healthcare).tw. | 8385 |
| 12 | or/4-11 | 304831 |
| 13 | patient care team/ or nursing, team/ | 72427 |
| 14 | INTERPROFESSIONAL RELATIONS/ | 53690 |
| 15 | (inter-professional* or interprofessional*).tw. | 14240 |
| 16 | (inter-disciplin* or interdisciplin*).tw. | 42705 |
| 17 | (inter-occupation* or interoccupation*).tw. | 12 |
| 18 | (multi-profession* or multiprofession*).tw. | 3090 |
| 19 | (multi-disciplin* or multidisciplin*).tw. | 108387 |
| 20 | (multi-occupation* or multioccupation*).tw. | 32 |
| 21 | (trans-discipinar* or transdisciplinar*).tw. | 2272 |
| 22 | (cross-occupation or crossoccupation).tw. | 1 |
| 23 | (cross-disciplin* or crossdisciplin*).tw. | 2095 |
| 24 | (cross-profession* or crossprofession*).tw. | 134 |
| 25 | Interdisciplinary Communication/ | 18201 |
| 26 | team*.mp. | 242826 |
| 27 | physician-nurse relations/ | 2414 |
| 28 | 13 or 14 or 15 or 16 or 17 or 18 or 19 or 20 or 21 or 22 or 23 or 24 or 25 or 26 or 27 | 401685 |
| 29 | 3 and 12 and 28 | 2959 |
| 30 | limit 29 to (english language and yr="2022 -Current") | **427** |

**Ebsco – CINAHL (English, 2022=>, excluding MEDLINE); n=577**

| S1 | TI (primary N3 team*) OR AB (primary N3 team*) | Search modes - Boolean/Phrase |  |
| --- | --- | --- | --- |
| S2 | (MH "Multidisciplinary Care Team") | Search modes - Boolean/Phrase |  |
| S3 | (MH "Team Nursing") | Search modes - Boolean/Phrase |  |
| S4 | (MH "Primary Health Care") | Search modes - Boolean/Phrase |  |
| S5 | (MH "Ambulatory Care") | Search modes - Boolean/Phrase |  |
| S6 | TI ( (outpatient N2 (care or service*)) ) OR AB ( (outpatient N2 (care or service*)) ) | Search modes - Boolean/Phrase |  |
| S7 | (MH "Family Practice") | Search modes - Boolean/Phrase |  |
| S8 | (MH "Group Practice") OR (MH "Joint Practice") | Search modes - Boolean/Phrase |  |
| S9 | TI (primary N2 care) OR AB (primary N2 care) | Search modes - Boolean/Phrase |  |
| S10 | TI (primary N2 healthcare) OR AB (primary N2 healthcare) | Search modes - Boolean/Phrase |  |
| S11 | S1 OR S2 OR S3 OR S4 OR S5 OR S6 OR S7 OR S8 OR S9 OR S10 | Search modes - Boolean/Phrase |  |
| S12 | (MH "Rural Health Personnel") OR (MH "Rural Health Centers") OR (MH "Hospitals, Rural") OR (MH "Rural Population") OR (MH "Rural Health Services") OR (MH "Australian Rural Nurses and Midwives") OR (MH "Rural Health Nursing") OR (MH "Rural Areas") OR (MH "Association for Australian Rural Nurses") OR (MH "Services for Australian Rural and Remote Allied Health") OR (MH "Rural Health") OR (MH "Frontier Nursing Service") | Search modes - Boolean/Phrase |  |
| S13 | (rural* or remote* or isolat* or north* or "sparse population" or "sparse populations") | Search modes - Boolean/Phrase |  |
| S14 | S12 OR S13 | Search modes - Boolean/Phrase |  |
| S15 | (MH "Interprofessional Relations") | Search modes - Boolean/Phrase |  |
| S16 | TI ( (inter-professional* or interprofessional*) ) OR AB ( (inter-professional* or interprofessional*) ) | Search modes - Boolean/Phrase |  |
| S17 | TI ( (inter-disciplin* or interdisciplin*) ) OR AB ( (inter-disciplin* or interdisciplin*) ) | Search modes - Boolean/Phrase |  |
| S18 | TI ( (inter-occupation* or interoccupation*) ) OR AB ( (inter-occupation* or interoccupation*) ) | Search modes - Boolean/Phrase |  |
| S19 | TI ( (multi-profession* or multiprofession*) ) OR AB ( (multi-profession* or multiprofession*) ) | Search modes - Boolean/Phrase |  |
| S20 | TI ( (multi-disciplin* or multidisciplin*) ) OR AB ( (multi-disciplin* or multidisciplin*) ) | Search modes - Boolean/Phrase |  |
| S21 | TI ( (multi-occupation* or multioccupation*) ) OR AB ( (multi-occupation* or multioccupation*) ) | Search modes - Boolean/Phrase |  |
| S22 | TI ( (trans-discipinar* or transdisciplinar*) ) OR AB ( (trans-discipinar* or transdisciplinar*) ) | Search modes - Boolean/Phrase |  |
| S23 | TI ( (cross-occupation or crossoccupation) ) OR AB ( (cross-occupation or crossoccupation) ) | Search modes - Boolean/Phrase |  |
| S24 | TI ( (cross-disciplin* or crossdisciplin*) ) OR AB ( (cross-disciplin* or crossdisciplin*) ) | Search modes - Boolean/Phrase |  |
| S25 | TI ( (cross-profession* or crossprofession*) ) OR AB ( (cross-profession* or crossprofession*) ) | Search modes - Boolean/Phrase |  |
| S26 | TI team* OR AB team* | Search modes - Boolean/Phrase |  |
| S27 | (MH "Nurse-Physician Relations") | Search modes - Boolean/Phrase |  |
| S28 | S15 OR S16 OR S17 OR S18 OR S19 OR S20 OR S21 OR S22 OR S23 OR S24 OR S25 OR S26 OR S27 | Search modes - Boolean/Phrase |  |
| S29 | S11 AND S14 AND S28 | Expanders - Apply equivalent subjects  Search modes - Boolean/Phrase | Results (642) |
| S30 | S11 AND S14 AND S28 | Limiters - Published Date: 20220101-20240530; English Language; Peer Reviewed; Exclude MEDLINE records  Expanders - Apply equivalent subjects  Search modes - Boolean/Phrase | Results (**577**) |
