# Supplementary material for: A scoping review of innovations that promote interprofessional collaboration (IPC) in primary care for older adults living with age-related chronic disease in rural areas
Source: PLoS One. 2025 Sep 3;20(9):e0331327. doi: 10.1371/journal.pone.0331327 (PMC12407450; doi:10.1371/journal.pone.0331327)
Supplement: S3 Appendix — (DOCX) [file pone.0331327.s003.docx]

**S3 Appendix. Charted data.**

**All charted data consists of verbatim extracts that may be paraphrased or summarized for length or clarity.**

| Author,  Year,  Country | Methods, Sample | - Rural or Rural-Urban - Province/State - Definition of Rural | Age-related chronic disease(s) (CD)  - main CD(s) of focus, and other comorbid CDs reported | Interprofessional collaboration (IPC) in primary care   - Definition of IPC - PCPs (primary care providers) involved   Specialist involvement | Innovation (Research Question #1) | Rural/Remote strengths and challenges that reportedly impacted the innovation (Research Question #2) | Benefits of the innovation (Research Question #3) |
| --- | --- | --- | --- | --- | --- | --- | --- |
| Liu et al. [21]  2022  United States | **Methodology:** Quantitative   - Interventional, single-arm without concurrent controls, longitudinal   - Montreal Cognitive Assessment   - Geriatric Depression Scale   - Caregiver/Patient Satisfaction Survey   - Provider Satisfaction Survey   - **Patient/Caregiver Follow-up:** - by navigator at 1-week via phone call, and at 1-month via text (satisfaction survey) - by geriatrician virtually at least every 6 months - **outcome assessments at 3, 6, and 12-months**   - Access to care   - Integration of services   - Caregiver burden   - Patient quality of life   - New dementia diagnoses   - 1-month and 3-month healthcare utilization (emergency, department visits, avoidable hospitalizations, readmission)   **Sample:** from 2 rural primary care clinics via referral by PCP   - **Patients/Caregivers:** 115 (n=105 CG satisfaction survey) - **Providers:** 18 (n=8 satisfaction survey)   **Sex:** not reported  **Age:** not reported (sample drawn from rural primary care clinics with previously identified *‘high numbers of patients age 65+ years’*)  **Race:** not reported | - **Rural** - **North Carolina** (counties of Stanly and Cleveland) - Participants were referred by PCP from ***‘two*** ***rural primary care clinics****’* | **Dementia** | **IPC:**   - team-based approach   **PCPs:**   - Initial referral by PCP (not specified; on-site) - Social Worker (Dementia Navigator) (off-site; virtual and via phone)   **Other personnel:**   - Medical Assistant (on-site)   **Specialist involvement:** Yes; Geriatrician (actively participated in virtual memory clinics) | **Virtual Memory Clinic** – practice-based virtual dementia screening and referral program using telehealth, adapted from an existing in-person, team-based version of the program in an urban center, to accommodate and improve access for patients living in rural settings. Involved patient assessment by geriatrician, access to dementia navigator/social worker, and follow-up care. | **Challenges:**   - **Availability of resources**   - Consideration of facility and staffing should be clearly assessed and communicated at program implementation. | **Benefits to Patients/Caregivers**   - **Continuity of care:** in addition to having virtual access to a geriatrician, patients/caregivers maintained continuity of care by having cognitive screenings performed by familiar primary care staff. Caregivers received monthly - **Education and Support:** Monthly phone calls from the navigator to build rapport, solicit questions, and provided non-pharmacologic interventions and resources   **Benefits to PCPs**   - The VMC provided **support in managing multiple aspects of routine care** (e.g., caregiver education, advanced care planning, driving safety evaluation, dementia-related behavioral management, and reduced triage communication for behavioral issues) |
| Chen et al. [22]  2022  China | **Methodology:** Quantitative   - Interventional, cluster randomized controlled trial, longitudinal   Conducted from January 1, 2014 through September 30, 2018).  **Baseline, 3, 6, 9, and 12 months**  **Primary outcomes:**   - - Hamilton Depression Rating Scale (Chinese translation)   - Hypertension control (based on BP)   **Secondary outcomes:**   - - Sociodemographic data at baseline   - Health-related quality of life (WHOQOL-BREF)   - Number of comorbidities (in EMR)   - Impairment in basic (ADL) and instrumental activities of daily living (IADLs)   - Medical Outcomes Study Social Support Survey Chinese version (MOS-SSS-C)   - Perceived social network size (Lubben Social Network Scale)   **Sample:**  From 2899 eligible residents in 218 rural villages (each with 1 primary care clinic) located in 10 randomly selected townships, **n=2365** clinic patients > 60 years with diagnosed hypertension and clinically significant depressive symptoms (as per the Patient Health Questionnaire-9 score of >10).  **Intervention Group:** n=1232 (from 102 villages)  **Comparison Group:** n=1133 (from 116 villages)  *Participants, primary care providers, and assessors were blinded to study objectives and hypotheses (but not to group assignment).*  **Age:** mean age 74.5 years  60-69 years: 783  70-79 years: 849  80-89 years: 665  > 90 years: 68  **Sex:** 1576 female, 789 male  **Race:** not reported | - **Rural** - Tonglu and Jiande counties of Zhejiang Province - *these 2 counties have a total of 920,000 residents distributed across 30 townships with a total of 602 villages. The health needs of each village are provided by a clinic staffed by 1 PCP without other nursing support. Mental healthcare is provided by 1 county-level mental hospital, and residents’ social needs are provided by the village’s Aging Association staffed by local residents* | **Comorbid Hypertension and Depression** | **IPC:**   - collaborative care - team   **PCPs:**   - Village Doctors (VDs) - Aging Workers (AWs) (‘Women workers’ and ‘Health liaisons’)   **Specialist involvement:**  Yes; Psychiatrist Consultants - psychiatrists from the county mental hospital provide consults to VDs when required, and travel to villages to conduct diagnostic assessment and in consultation with the VD to initiate antidepressant Rx when needed. | **Chinese Older Adult Collaborations in Health (COACH) 12-month team-based collaborative care intervention** to treat older rural adults with co-morbid hypertension and depression, compared to Enhanced Care-as-Usual (eCAU, refer to as “enhanced” because providers were told when their patients screened positive for depression, and were provided with copies of antidepressant treatment guidelines). | none reported | **Benefits to Patients and Providers:**  Significant improvements in both depression symptom severity and hypertension control rates. |
| Partogi et al. [23]  2022  United States | **Methodology:** Quantitative   - Interventional, 3-phase design with randomized, 3-month prospective pilot trial - **Baseline only**   - Demographics, medications, BP - **During and Post-**    - encounters with nonphysician HCPs (as measurement of proportion of V4 cardiovascular guideline recommendations delivered) - **Post-**   - Patients (n=154) and HCPs interview or survey via phone or web-based   **Samples:**  **Phase 1:**  Multidisciplinary team members (n=35)  **Interviews** (n=18) purposive sampling of patients, HCPs, administrators, institutional leadership and 8 patients (mean age 77, SD 10 years; n=6, 75% women and n=2, 25% men).  **Ideation workshops (2):** HCPs including 5 RNs, 4 NPs, 3 LPNs, 2 pharmacists, 3 physicians, 1 tobacco cessation coach, and  1 care coordinator.  **Phase 2:**  **Prototyping workshops**  4 RNs, 4 NPs, 3 LPNs, 2 pharmacists, 1 tobacco  cessation coach, 1 RN care coordinator, and 3 physicians.  **Detailed workshops** for nurses (4 NPs, 3 LPNs, 4 RNs, and 1 RN  care coordinator), pharmacists (n=2), and tobacco cessation  coaches (n=1).  **Experiments in clinic** – various combinations HCPs  **Phase 3:**  **Pilot trial: 369 patients** from a Cohort Knowledge Solution ASCVD registry with ASCVDs on ≤3 *V4* guideline recommendations  Control Group: n=182  Intervention Group: n=187  **Sex:**  CG: n=92 male  IG: n=95 male  **Age:** mean age 71 years  CG: +/- 13 years  IG: +/- 14 years  **Race:**  CG: n=167 White; n=171 ‘not Hispanic or Latino’  IG: n=95 White; n=174 ‘not Hispanic or Latino’ | - **Rural** - Mower County, Minnesota - *Austin is the only urban core area in Mower County, and has an urban core of 25,000* *residents* where **rural counties were defined as *those with an urban core of ≥10,000 to <50,000 people*** [as in Harrington et al. Call to action: rural health: a presidential advisory from the American heart association and American stroke association. Circulation 2020 Mar 10;141(10): e615-e644] | **Atherosclerotic cardiovascular diseases (ASCVDs)** | **IPC:**   - team-based care   **PCPs**   - Physicians - Nurse Practitioners - Physician Assistants - Registered Nurses - Licensed Practical Nurses - Pharmacists - Tobacco cessation coaches - Other nursing teams such as care coordinators, complex disease coordinators   **Other**  **Professionals:**   - Administrators, institutional leadership - Design, IT, and clinical informatics teams (centralized Rochester)   **Specialist involvement:** No | **Rural-specific team-based care model** for the coordination and delivery of tailored preventive cardiovascular care to patients with ASCVDs assisted by clinical decision support (CDS) technology, and the creation of redesigned workflows and a novel CDS tool. | **Strengths**   - Patients and providers **preferred delivery of care by local providers** which was reportedly ideal for this rural-specific intervention where rural **social networks are often ‘tightly knit’.** | **Benefits to Patients**   - **the proportion of patients who saw advanced practice providers** (nurse practitioners and physician assistants), **pharmacists, or tobacco cessation coaches for the delivery of guideline recommendations in the intervention arm was greater than that in the control arm**. - **Flexibility in consideration of patient preference for visit type** mitigated barriers to transportation and limited mobility   **Benefits to Providers**   - **Participatory design and computer-supported participatory work theories** drove the collaborative design that allowed for the translation of rural HCP’s needs into technical requirements and were **key design characteristics** for sustainability.   **Benefits to Patients and Providers**   - A novel team-based model of care **specifically designed for the delivery of care in resource-constrained clinics located in rural communities** and promoted teamwork with shared responsibilities among team members. Regional hubs could benefit clinics that lack certain HCPs by having HCPs from other rural clinics provide support remotely when a specific expertise is unavailable. - **New tools and processes improved identification of ASCVD patients with gaps in preventive care**, and those gaps were addressed with appropriate providers and tailored interventions. - **The pilot trial demonstrated that this model** (1) connected the **right HCP with the right patient**, (2) **saved time** by reducing the need for manual chart review, (3) **enabled HCPs** to work to the top of licensure, (4) **potential for expansion** to other conditions, and (5) **promoted interdisciplinary collaboration** to optimize care. - The **CDS were populated with data automatically extracted from EHRs**, which enabled the delivery of longitudinal care for patients with ASCVDs, and was integrated within provider workflows. - **Flexibility in consideration of patient preference for visit type** **enabled a patient-centered health connection** beyond traditional symptom-based visits |
| Disler et al. [24]  2022  Australia | **Methodology:** Quantitative   - Observational, retrospective, cross-sectional   - Analysis of all patients screened for dementia between 2014 and 2018, referral rates, and referral characteristics   - Demographics including SEIFA (Socio-Economic Indexes for Areas) rank, medical history, smoking status   - Clinical and neuro exam, bloodwork, renal and hepatic function tests, assessment for diabetes, thyroid function, vitamin B_12_ and folate, brain CT   - Mini-Mental State Examination (MMSE), GP Assessment of Cognition (GPCog), and Geriatric Depression Scale (GDS).   - Referral status & outcomes (for geriatrician, psycho-geriatrician, or both)   **Sample: 818 patients** over 65 years of age from a group of 7 general practices  **Age:** mean age 77.37 years  **Sex:** 418 female, 398 male  **Race:** 3 (of 557) Aboriginal and Torres Strait Islander | - **Rural-regional** - *Loddon-Mallee region of Victoria, Australia with a catchment area of 12,213 km^2^* *and 58,602 people over age 65 years* (cited by authors as in Department of Health and Human Services. Loddon Mallee Region 2015. In: Victoria DoHaHS, editor. Victoria, Australia: Department of Health and Human Services Victoria; 2017) | **Dementia**  Reported medical history of:   - Diabetes - Stroke - Cardiovascular disease - Depression   Anxiety | **IPC:**   - Multidisciplinary, collaborative   **PCPs:**   - GPs - Practice Nurses (PNs)   **Specialist involvement:** Yes; GP access to specialist advice, both through the co-location of a geriatrician and psycho-geriatrician specialists consulting within the primary setting, and through informal as-needed dialogue either in person or on the telephone. | **A new community-based GP-led multidisciplinary dementia model of care** that incorporates provider education/training, standardized tools for early screening/detection, and a referral pathway to a community-based specialist. | none reported | **Benefits to Patients and Providers**   - **Validated screening tests were conducted on approximately 24.2% of all patients over age 65** who presented at the general practices, most of which would not have likely been formally assessed before program implementation. - **68.9% (564/818) of patients screened triggered a referral** to specialist and of those, **171 successful referrals** to a geriatrician and/or psycho-geriatrician were made. |
| Zupa et al. [25]  2022  United States | **Methodology:** Quantitative   - Interventional, pre-post and retrospective chart review   - Demographics and baseline medications   - Baseline and 12-months post- enrollment A1C, LDL, systolic BP, weight, vitals   - EMR chart review of A1C levels 6 and 12-months before the intervention (for historical comparison)   **Sample:** 87 Veterans with type 2 diabetes (T2DM) from 2 rural primary care sites (Erie and Butler).  Erie: n=37  Butler: n=50  **Sex:**  Erie: n=37 male  Butler: n=48 male  **Age:**  Erie: 68.9 years (SD 6.4)  Butler: 65.9 years (SD 10.3)  **Race:**  Erie: n=35 White  Butler: n=44 White | - **Rural** - Western Pennsylvania (Butler and Erie) - Rural primary care | **Diabetes**  Also reported:   - Hypertension - Hyperlipidemia - Mental health diagnosis - Nephropathy - Coronary artery disease - Stroke - Peripheral arterial disease | **IPC:**   - Multidisciplinary collaborative team-based care   **PCPs:**   - Certified Registered Nurse Practitioner (CRNP) (DCN liaison) - Clinical Pharmacy Specialists (CPS) (DCN liaisons) - Registered Nurses - Registered Dietitian - Home-based primary care providers - Home telehealth (HT) coordinators   **Specialist involvement:** Yes; a centrally located team of diabetes specialists (endocrinologists, CRNPs, and diabetes care and education specialists (DCES)) | **Diabetes Care Network (DCN) -** a novel model to disseminate team-based diabetes specialty care in the rural primary care setting, developed using the chronic care model (CCM).  **A technology-enabled remote collaborative care partnership** between centrally located diabetes specialist care teams and rural primary care clinical liaisons with a 6-week period of **remote self-management education and support and medication management, and subsequent comanaged care** for the remainder of the 12-month intervention. | none reported | **Benefits to Patients and Providers**   - **Enhanced access to multidisciplinary, collaborative guideline-based diabetes specialty care provided at the local level** in the rural primary care setting, including diabetes self-management education and support (DSMES) and pharmacologic management. - **Remote technology and embedded clinical liaisons** within the existing rural primary care setting allowed for **specialist involvement** with **no travel** to a larger center required, and PCPs have the **additional support** of the specialist team when needed. - **Overall improvements** in A1C, LDL, and systolic blood pressure. Even patients already receiving diabetes care within the PACT structure had significant improvements in A1C during this intervention. |
| Camargo et al. [26]  2021  Brazil | **Methodology:** Quantitative   - Observational, cross-sectional (diagnostic accuracy/evaluation)   - Questionnaire (demographic/socioeconomic data)   - Anthropometric   - Blood Pressure   - Capillary blood glucose   **Sample:** 913 people with hypertension and/or diabetes living within coverage areas of 4 rural basic health units (BHU), who attended routine primary care team activities at health fairs and in patient groups  **Sex:** 606 females, 307 males  **Age:**   \| **Years** \| ***n*** \| \| --- \| --- \| \| < 40 \| 61 \| \| 40–59 \| 358 \| \| **60–79** \| **425** \| \| **> 80** \| **69** \|   **Race:** 179 White, 454 Brown, 234 Black, 46 Other | - **Rural** - **Communities in a Northeastern Municipality Northeastern Brazil** - Municipality Total Area: 3,254,186 km2, estimated population 338,480 (approx. 12% rural); rural area in this study were 4 of 18 PHC BHUs covering entire rural population; furthest rural unit is 82 km from municipal laboratory | **Diabetes and**  **Hypertension** | **IPC:**   - primary care teams - family health teams   **PCPs:**   - Doctor - Nurse - Community health worker(s)   **Specialist involvement:** No | **Point-of-care device to measure glycated hemoglobin (HbA1c).**  Use of a point-of-care device during routine rural PHC team activities to measure capillary glycemia glycated hemoglobin (HbA1c) to assess glycemic control and identify new diabetes diagnoses, to improve access and eliminate distance barrier for rural folks.   - **Community health workers** extended invitations to patients during home visits   **Physicians and nurses** provided education and assessments, patients with higher than threshold values were referred to physician, and those with positive or high HbA1c level were to repeat test within 3 months | **Challenges:**   - **Rurality** (‘distance from urban center’) **was a distance barrier negatively affected access to diabetes services** - *Although study areas were covered by the family health program, each with a primary care unit located within, the distance barrier in terms of the location of the Municipal laboratory renders the efforts of the rural health teams insufficient to ‘identify individuals with diabetes, those with high potential for the development of diabetes, or those with inadequately controlled blood glucose levels’* | **Benefits to Patients**   - **Improved access:** Use of point-of-care devices for HbA1c level measurement **improved access** for people living **in rural or remote areas**   **Benefits to both Patients and PCPs**   - **Feasibility/Ease:** Rural PHC providers were **able to use** the testing device with their patients **during routine care activities** - **Effectiveness:** Resulted in **increased rates of detection** with new diagnoses and identification of uncontrolled glycemia (*in a health system that had previously been unsuccessful at regularly monitoring HbA1c levels*) |
| Lu et al. [27]  2021  United States | **Methodology:** Quantitative   - Interventional, retrospective, longitudinal, non-randomized   - HbA1C level   - Secondary outcomes for diabetes quality indicators including:   - BP control  - statin use  - angiotensin-converting enzyme inhibitors or angiotensin II receptor blockers use, and  - annual urine microalbumin measurement  **Sample: 9010 patients (veterans) with diabetes who received V-IMPACT PHC services Jan/18 to Dec/19**, identified from outpatient VA clinical and administrative database.  **Rural:** 6721 (75%)  ***Propensity score matched***  **Intervention Group (n=4505)**  **Sex:** 4309 (95.6%) male  **Age:** mean age 68.4 (+/- 10.3) years  **Race:**  77% White  12% Black  3% Hispanic  11% Other & Unknown  **Rural:** 3372 **Urban:** 1133  **Control Group (n=4505; usual care)**  **Sex:** 4321 (96%) male  **Age:**  mean age 68.2 (+/- 10.9)  **Race:**  77% White  12% Black  3% Hispanic  11% Other & Unknown  **Rural:** 3349 **Urban:** 1156 | - **Rural – Urban (primarily rural)** - Multi-site (National/USA) - VA **rurality codes** as per the Rural-Urban Commuting Areas (RUCA) system | **Diabetes**  Reported co-morbidities:   - Atrial fibrillation - Alcohol use - Cancer - Congestive heart failure - Chronic kidney disease - COPD - Cardiovascular disease - Connective tissue disease - Depression - Hypertension - Liver disease - Peripheral arterial disease - PTSD - Peptic ulcer disease   Sleep apnea | **IPC:**   - team-based care   **PCPs:**   - Hub team (remote):   - Physicians  - nurse coordinators  - pharmacists  - social workers   - Clinic team:   - nurses  - medical assistants  **Other professionals:**  - (clinic) clerks  **Specialist involvement:** No | **V-IMPACT initiative**  involves a team-based model of primary care for patients via synchronous in-clinic videoconferencing between patient and clinic PCPs, and remote hub PCPs.   - V-IMPACT model (Virtual Integrated Multisite Patient Aligned Care Team (VIMPACT), clinic-to-clinic synchronous videoconferencing (patient and PCPs together in clinic videoconference (with digital stethoscopes/high-def camera/trained nurses) with hub PCPs. | **Challenges:**   - Known rural disparities in diabetes care might be reflected in **lower absolute percentages of patients who met quality indicators** in this study. - Though V-IMPACT was not offered exclusively to rural sites, **clinics in rural areas were more likely to experience these needs and utilize V-IMPACT** as a result which leverages telemedicine to virtually reallocate available providers in urban areas, **helping to close short-term supply gaps and meet existing needs**. | **Benefits to both Patients and PCPs in Rural Communities**   - **V-Impact helped close a gap in provider shortages** where clinics opted to participate in V-IMPACT when they had to fill unexpected provider vacancies or meet demand of growing patient population. - V-IMPACT video visits included **high-definition cameras, vital signs, and a facilitator** for physical exams, providing high quality care   **Benefits to Patients**  ***•* Familiarity and comfortability** with local primary care clinic, staff and providers  • In-clinic telemedicine **overcomes technology and connectivity barriers**  • **Continuity of Care is strengthened** by hub clinicians visiting spoke clinics quarterly for in-person examinations and routing procedures*.*  *•* **Higher percentages of V-IMPACT patients met quality metrics**, which could be related to having more annual PC visits, or more resources/providers available within each hub. |
| Thanachayanont et al. [28]  2021  Thailand | **Methodology:** Quantitative   - Observational, prospective, cohort (with retrospective comparison group)   - **Estimated glomerular filtration rate [eGFR]**   - Serum creatinine   - Fasting blood sugar   - Low-density lipoprotein   - Haemoglobin A1c [**HbA1c**]   - **Haematocrit**   - Serum potassium, **serum bicarbonate**, serum albumin   - Proteinuria   - **Blood pressure**   - **Use of NSAIDs or herbs**   **Sample: 914 (**813 for entire study period) Stage 3-4 CKD patients (from a pool of 1211 patients with CKD Stage 3-4)  **Sex:** 609 (67%) female  **Age:** mean age 62 (+/- 6) years  **Race:** not reported  **Comparison Group:** from previous intervention study in less community-level setting, from which this study stemmed, ESCORT-1 control group (n=201), and propensity score matched at a ratio of 1:3, group (n=144/201) | - **Rural** - **Kamphaeng Phet Province** - **400 km north of Bangkok**, where each of 5 districts have one 30-90 bed hospital providing primary and simple secondary care to 20K to 60K residents per district, with smaller subdistricts and village health centers within (approx. 3K-4K villagers in each subdistrict) | **Chronic Kidney Disease (CKD)**  Reported co-morbidities:   - Hypertension - Diabetes - Hyperlipidemia - Hyperuricemia - Cardiovascular disease - Cerebrovascular disease | **IPC:**   - multidisciplinary care team   **PCPs:**  HMDT   - GPs - Chronic disease nurse manager - Nutritionist - Pharmacist - Physical therapist   CCN   - Community nurses - Community health volunteers   **Specialist Involvement:** No | **ESCORT-2** (Effectiveness of Integrated Care on Delaying Progression stage 3–4 Chronic Kidney Disease in Rural Communities of Thailand):  **A community-level Multidisciplinary integrative model of care for monitoring CKD progression** in local, routine rural PHC, with a variety of PCPs and a combination of clinic and home visits.   - **integrated care model** consisting of a **HDMT** (hospital **multidisciplinary team**) visit every 3 months (included medical care, education, and live demonstrations)   **and**   - **home visits by CCN** (community care network) team every 6 months (included BP, dietary assessment, and general health advice) | **Challenges:**   - **Low resource availability was reported as a limitation in this rural intervention where** *‘due to a large number of patient load with respect to the number and time allowable of the health personnel of each community hospital or each sub-district health office, individual case education and counselling were quite limited and group education had to be utilized instead. This might have had some impact on the final outcome.’* | **Benefits to Patients**  ***•* Better control of CKD progression**  • **Positive impact on quality of care** with regular home visits and comprehensive, multidisciplinary team-based care  *•* **Effective decline of eGFR overall**, and beneficial effects of close monitoring on other biochemical parameters  *•* **Education** on lifestyle modification, medication adherence, and BP control likely contributed to positive patient outcomes |
| Wopat et al. [29]  2021  United States | **Methodology:** Quantitative   - Interventional, single-arm without concurrent controls, pre-post   - **Rates of DXA scanning completion**   - completed DXA scans   - new diagnoses of osteoporosis or osteopenia   - patients eligible for treatment based on DXA scan results   - patients who started oral bisphosphonate therapy   **Sample: 232** rural veterans aged 70+ receiving primary care from a Patient-Aligned Care Team (PACT)  **Sex:** 232 male (100%)  **Age:** *‘average’* age 76 years  **Race:** not reported | - **Rural** - **Wisconsin** - *‘Rural and highly rural were defined using the Rural-Urban Commuting Area codes’ (MacKinney et al. 2014 - Access to rural health care - a literature review and new synthesis. RUPRI health panel.)* | **Osteoporosis**  Reported co-morbidities:   - Rheumatoid arthritis - Chronic liver disease - Previous fracture | **IPC:**   - interprofessional - collaborative - team care delivery   **PCPs:**  PACT includes:   - **Physician** - Nurses - **Clinical Pharmacy Specialists** (and student pharmacists) - Registered dietitians - Social Workers   **Other professionals** (in PACT):   - Schedulers   **Specialist Involvement:** No | As part of the Patient-Aligned Care Team (PACT), Pharmacist-led  Osteoporosis Risk Assessment and Treatment for Improvement in Fracture Rates in the Elderly **(PhORTIFy) is** **a** coordinated, pharmacist-led DXA **osteoporosis screening and treatment service** using student pharmacists to identify and appropriately treat patients**.** | none reported | **Benefits to Patients**   - Using **dedicated resources** **and time** to provide **targeted population outreach**, instead of screening/managing osteoporosis at a standard appointment with other health conditions - **Filled a gap** where nearly half of those screened were newly diagnosed and eligible for Tx   **Benefits to Providers**   - Involving student pharmacists in the intervention while not essential enhanced their learning and **increased efficiency** by reducing the time [clinical] pharmacists spend on screening tasks and allowing them more time to focus on Tx and other duties |
| Woodham et al. [30]  2020  Thailand | **Methodology:** Quantitative   - Interventional, non-randomized, pre-post - **Baseline only**   - STEPS Questionnaire (WHO STEPwise approach to noncommunicable disease surveillance) - **Pre-Post**   - Medication Adherence   - Height/Weight/BP   - Estimated glomerular filtration rate (eGFR)   **Sample:** 200 elderly persons with hypertension (from village zone one under the administration of the Noonsomboon Health Center)  **Intervention Group (n=100)**  **Sex:** 74 female, 26 male  **Age:** mean age 67.49 (+/- 6) years  **Control Group (n=100; usual care)**  **Sex:** 71 female, 29 male  **Age:** mean age 66.88 (+/- 5.7) years  **Race:** not reported | - **Rural** - **Buengkan Province** - *‘a low-income,* ***remote area*** *on the Thai–Laos border in northeast Thailand’* where access to health facilities is limited due to distance from villages and a lack of any or affordable public transportation | **Hypertension**  Reported co-morbidities:   - Diabetes - Heart disease - Kidney disease | **IPC:**   - Multidisciplinary team   **PCPs:**   - Nurses - Public Health personnel trained in pharmacy - Community health volunteers   **Specialist Involvement:** No | Community-level, multidisciplinary, **multicomponent,** team-based, ‘patient-centric’ **hypertension intervention to improve BP control**, via screening, prescription refilling, BP measurements, group education, home-based care (when required), and comprehensive home visits with individual guidelines for long-term lifestyle modifications, over a 3-month period. | none reported | **Benefits to Patients**  • **Better BP control, medication adherence, and maintenance of kidney function (eGFR)**  • **Accessible, multi-disciplinary, team-based, multi-component, *‘patient-centric’*, feasible, effective,** intervention integrated into community-level routine care close to their home |
| Burge et al. [31]  2019  United States | **Methodology:** Quantitative   - Observational, biphasic, longitudinal (retrospective randomized chart review, prospective nonrandomized assessment)   - Descriptive demographic characteristics and main outcome measures **(Patient Health Questionnaire) PHQ-2 (short-form) and PHQ-9, screening rates (over 3 cycles @ 3 unspecified timepoints between January 2014 and December 2017)**   **Sample (convenience):**  **From 4 primary care rural health clinics,**  Initial convenience sample for records of 50 patients age 65+ years Dx with anxiety or depression Jan/14 to Dec/17 (those with no known chronic illnesses were excluded)  Cycle 1 (**n=50 patients**):  **Age:** range 65 to 99 years, 54% were aged 65-74 years  **Sex:** 32 female, 18 male  Cycle 2 (**n=14 patients screened in Cycle 1**): from 11 days of screening eligible patients, chart review  **Age:** 65 to 89 years, 57% age 65-74 years  **Sex:** 9 female, 5 male  Cycle 3 (**n=153 patients**): full system implementation and measurement of PHQ-9 & PHQ-2 screening rates  **Age:** 65 to 99 years, 45.7% aged 65-74 years  **Sex:** 96 female, 57 male  **Race:** not reported | - **Rural** - **Nebraska** (2 counties in south-central) - 6,850 people living within 1,262 square miles; counties defined as ‘frontier’ (population density of 5.6 - 6.5 people per square mile) | **Depression**  and  comorbid chronic conditions:  endocrine/nutritional/metabolic disease, mental/behavioral disorders, nervous system, eye/adnexa, ear/mastoid, circulatory system, digestive system diseases, skin/subcutaneous tissue diseases, musculoskeletal and connective tissue diseases | **IPC:**   - Interprofessional small-team approach   **PCPs:**  **Across 4 rural health clinics:**   - Advanced practice Nurses - Physician - Nurses - Medical assistant   **Other professionals:**   - Reception staff - Director of Nursing Informatics - Director of Nursing - Chief Executive Officer   **Specialist Involvement:** No; referral only | Development and implementation of **an EMR-supported biphasic secondary depression screening process** embedded into primary care clinic check-in processes.   - People aged 65+ years scoring positively on PHQ-2 triggered to complete PHQ-9 on same day **during same clinic** visit so provider can **at the same time** consider Tx initiation or changes during that visit. | **Challenges:**   - *Reported health department statistics indicating an existing regional gap for Nebraska in primary care for men* - *‘autonomy is highly valued by rural people and creates an impact on the use of health care services’ and perhaps participation in this study* - **PHQ-9 rates did not significantly improve likely** because *screening process lacked connection to EMR use and* ***relied on*** *PHQ-2 screening to prompt* ***in-person communication*** *between nursing and health care provider,* ***and*** *in-person communication* ***often failed*** *to notify physician of need for further assessment* | **Benefits to Patients & PCPs:**   - Initial randomized convenience chart review of 50 eligible patients showed **zero *depression screening documented in the EMR*** *which is compelling evidence in support of the need for system change, where*   EMR-supported **PHQ-2** embedded depression **screening increased significantly** for both the screened initial group (n=14) and larger screened group (n=153), and half of the 14 eligible initially screened scored positively on the PHQ-2 |
| Zheng et al. [32]  2019  China | **Methodology:**  Quantitative   - Interventional, nonrandomized, pre-post - **Baseline only**   - Demographics, medications, Dx with diabetes or CHD - **Pre-Post**   - Medication Adherence   - Height/Weight/BP   **Sample:** 6575 hypertension patients, from a pool of 8139, from 24 PHC institutions (multistage stratified random sampling for study sites, from across each region in China)  **Sex:** 3896 female, 2679 male  **Age:** mean age 64.6 (+/- 10.4) years  **Race:** not reported  **Rural:** 2355 **Urban:** 4220 | - **Rural-Urban** - *2 provinces were selected from each region (3), and from those, one urban and one rural area were randomly selected* ***based on their social and economic development levels and populations****.* | **Hypertension and**  **Diabetes (as comorbid conditions and**  **at risk for cardiovascular disease (CVD))** | **IPC:**   - touted more as a GP intervention but clearly states ‘interventions **[delivered] via a team’** and some collaboration is involved   **PCPs:**   - GPs - Nurses - Public health doctors   **Specialist Involvement:** No | **One-year community-based hypertension management intervention** delivered to primary care patients with hypertension over one year. **One physical exam and at least 4 in-person follow-ups** and further interventions depending on outcomes. **Each visit included a physician and nurse, and ‘several essential interventions’:** Bp, symptom assessment, medication and/or lifestyle modification compliance, and prescriptions and/or more information as needed. | **Challenges:**  Greater intervention effectiveness for rural versus urban might be related to improved access to hypertension management, particularly for rural patients, within their community | **Benefits to Patients:**  • GPS were able to detect and address other health problems at earlier stage  • Overall mean reduction of 4.6 mmHg in systolic blood pressure (SBP) and 1.9 mmHg in diastolic blood pressure (DBP)  • Overall hypertension control rate increased from 44.4% to 59.1%  • Overall **more effective in rural versus urban areas**.  • **BP reduction after 1-year** intervention was **greater in rural patients** compared to urban, 6.6 mmHg versus 3.4 mmHg for SBP and 2.6 mmHg versus 1.6 mmHg for DBP, respectively.  • **Hypertension control rate** was higher before and after for urban compared to rural, but **increased more in rural** (22.1%) than urban areas (10.6%) after the one-year intervention, and increased across all 3 regions.  • **Age, BMI, region and being urban or in eastern or central regions** had a significant negative association with the reduction of SBP (P < 0.05).  **Benefits to Providers:**  • GPS were able to detect and address other health problems at earlier stage  • GPs could access professional training during the intervention |
| Acharya et al. [33]  2019  Australia | **Methodology:**  Quantitative   - Interventional, nonrandomized, pre-post (focus on proof of concept pilot study) - **Baseline, and 6 months** - HbA1c - BP - Cholesterol - BMI - Weight - eGFR - Urine ACR - Patient Activation Measure (PAM)   **Sample (pilot study only):**  from 14 practices, n=344 patients  Note: regional data from large-scale implementation of 1400 patients from 80 practices not included here due to no ages reported, and *‘significant variation in clinical processes and outcome measures’* and missing outcome data)  **Age:** mean age 63.2 years  **Sex:** 175 male (50.9%)  **Race:** not reported | - **Rural-Urban** - Hunter New England Local Health District in New South Wales - Includes *‘910,000 residents over 131,000 km area’*. Although this district includes a metropolitan city, study authors consider it a ***“large health district challenged with significant rural and remote geography and limited specialist workforce”.***   Thus data is non- disaggregated rural-urban data, but the district itself has ***“significant rural and remote geography”.*** | **Diabetes**  Also reported:   - Peripheral vascular disease - CVD - Cerebrovascular disease - Chronic kidney disease (CKD)   Retinopathy | **IPC:**   - Collaboration (amongst primary care team members, and with specialist teams).   **PCPs:**   - Physician (GP) - Practice Nurse (PN) - Diabetes Educator (DE)   **Specialist Involvement:** Yes; Endocrinologist provided feedback on practice diabetes patient data and performance feedback to GPs and PNs, and was part of initial patient case-conference, but not in follow-up unless needed. | **Diabetes Alliance Program (DAP) Pilot – a 4-part Quality-Improvement project:**   - Whole practice diabetes data analysis and performance feedback by endocrinologist. - 3-day case-conferences (GP, PN, DE, endocrinologist; 40 mins with 10 patients per day conducted in usual practice setting). - Structured diabetes educational programs for primary care clinicians.   Regional aggregate diabetes-related data monitoring including metabolic parameters, changes in clinical processes (such as Rx, referrals), and patient experience. | none reported | **Benefits to Patients**   - Overall, HbA1c levels showed highly signiﬁcant improvement from 60.0 +/- 16.2 to 55.3 +/- 12.6 mmol/mol (P < 0.001) - Weight improved from 95.5 +/- 20.9 to 94.5 +/- 21.5 kg (*P* = 0.006); and systolic BP 134 +/- 18 to 131 +/- 17 mmHg (*P* = 0.004). - Absolute 5-year cardiovascular risk improved from 18.4 (9.9 –30.6) to 16.7 (8.5 – 28.6) % (*P* < 0.001). - Patients reported feeling involved, comfortable and supported. - PAM scores improved with patient improvement in knowledge and conﬁdence in diabetes management.   **Benefits to PCPs**   - Practice nurses and GPs reported increased treatment competency and confidence. - Most GPs expressed very high satisfaction. - ‘Wider benefits’ included partnership/trust between specialist and primary care and timely referral to specialist services as needed. |
| Litke et al. [34]  2018  United States | **Methodology:** Quantitative   - Interventional, retrospective, longitudinal (quality-improvement evaluation)   **29-month evaluation period**   - Changes from baseline in HbA_1C_, BP values, statin Tx - Percentage of patients on goal lipid treatment at discharge - Percentage of patients who quit tobacco use   **Sample: n=554 unique individuals** (rural veteran patients in the Northwest regional V-IMPACT Hub) that were consulted by a CPS (clinical pharmacy specialist) for diabetes, hyperlipidemia, hypertension control, or tobacco cessation from October 2014 through March 2017 and discharged from CPS management.  Note: 554 ‘unique’ individuals; total number of patient outcomes analyzed was greater, where more than 3400 CPS visits were completed.  For the first analysis, patients consulted by CPS for diabetes and/or hypertension were categorized into discharged at goal and discharged for any reason.  For the second analysis, all patients referred via provider consultation to CPS for diabetes or hypertension or hyperlipidemia, that that were seen by a CPS at least once.  **Sex:** ‘predominantly male’  **Age:** mean age 62 years  **Race:** not reported | **Rural veterans**   - **Rural – Urban (primarily rural)** - Multi-site (National/USA) - Refers to Veteran Affairs (VA) **rurality codes** as per the Rural-urban Commuting Areas (RUCA) system   The Northwest regional V-IMPACT Hub, based in Boi-  se, Idaho [urban], uses CVT and phone calls to  deliver primary care to **rural** **veterans**   - **throughout Alaska, Washington, Oregon, Idaho, and western Montana.** | **Diabetes**  **Hyperlipidemia**  **Hypertension** | **IPC:** team-based care  **PCPs:**  V-Impact Hub team (remote):   - Physicians - Physician Assistants - Nurse Practitioners - Clinical pharmacy specialists (CPSs)   **V-Impact Clinic team** (onsite with patient):   - Registered Nurses - Licensed Practical Nurses   **Other** (location not reported)   - Medical support assistants   **Specialist(s) involvement:** Yes   - Psychologists - Licensed clinical social workers   Psychiatrists | A **chronic disease management program** in primary care **with clinical pharmacy specialists** (CPSs), as part of the primary care team, provided comprehensive medication management services from October 2014 to March 2017, within the **V-IMPACT** Hub (Northwest regional Virtual Integrated Multisite Patient Aligned Care Team) that uses **clinical video telehealth (CVT) and phone calls** to deliver primary care services to rural veterans. | **Challenges:**   - The population in this study (mainly male armed forces veterans over age 60 years living in rural areas) was reported as a typically difficult population to engage in regular healthcare. - Rates of loss to follow-up were high and authors suggest that may have been due to various reasons, among which included telecommunication difficulties in rural areas. | **Benefits to Patients**   - Increased access to healthcare for veterans in rural and highly rural areas, via both telehealth and in particular, phone appointments which most patients preferred, and allowed patient and provider greater flexibility for scheduling and minimize travel burden (involved with telehealth). - Improved outcomes in veterans with diabetes, hyperlipidemia, and hypertension (and tobacco users). - Patients in both the diabetes and hypertension groups had a significant mean absolute HbA1c reduction and mean systolic BP reduction of 26.00 mm. - 93% of the hyperlipedemia group, were discharged on a lipid-lowering medication. - Tobacco cessation was achieved in 42% (of targeted patients).   **Benefits to Providers**   - Greater flexibility for scheduling appointments. |
| Jiamjariyapon et al. [35]  2017  Thailand | **Methodology:** Quantitative   - Interventional, cluster randomized controlled trial, prospective longitudinal   **Baseline, and 3-month intervals over 24 months** (June 2011 to July 2013)  **Data collected** included past medical history, physical exam, medications, and blood and urine samples.  **Primary Outcome:**   - **eGFR** (estimated Glomerular Filtration Rate) measured by the CKD-EPI equation (Chronic Kidney Disease Epidemiology Collaboration creatinine-based equation)   **Secondary Outcomes:**   - Lab parameters (such as BMI, BP, hemoglobin, HbA1C, LDL-C, Triglyceride, urine protein-creatinine, and 24-h urine Na and nPNA) and incidence of clinical endpoints including mortality, cardiovascular events, ESRD (End stage renal disease where eGFR is less than 15 ml/min/1.73 m^2^), and 50% increase in serum creatinine from baseline. - Quality of life ( SF-36, Thai version)   **Sample:** n = 442  2/11 districts were randomly selected. From those 2 districts 442/586 patients assessed for eligibility (stage 3 or 4 CKD) ‘were randomly selected to the study’ **Intervention group (IG):** 234 **Control group (CG): 208**  **Age:**  **IG:** mean age 62.3 years (+/- 6.4) years  **CG:** mean age 62.4 (+/- 7.9) years  **Sex:**  **IG:** 170 female  **CG:** 152 female  **Race:** not reported | - **Rural** - Two of eleven districts in Kamphang Phet province *located 400 km north of Bangkok.*   *resource limited setting* | **Chronic kidney disease (CKD) with Diabetes and/or Hypertension**  and any history of Ischemic Heart Disease (IHD)  Cerebrovascular accident (CVA) | **IPC:**   - multidisciplinary care team - collaborative   **PCPs:**   - 2 General Practitioners - 2 Chronic Care Nurses - 1 Pharmacist - 1 Nutritionist - 1 Physical Therapist - 2 Community Care Network teams (CCNs)   - 1 subdistrict health care officer   - 3 to 5 village health volunteers (VHVs)   - Selected patient family members   **Specialist involvement:** No | **ESCORT** (Effectiveness of Integrated Care on Delaying Progression stage 3–4 Chronic Kidney Disease in Rural Communities of Thailand), **a two-year community-based integrated care program** (ICP) with collaboration among and between a multidisciplinary care team and community care networks. | **Strength:** The use of community volunteers to address a shortage of health workers is well established in Thailand and other developing countries. Collaborative efforts between healthcare providers and non-healthcare members yielded similar or better clinical benefits. | **Benefits to Patients**   - Slowed the rate of eGFR decline and improved several clinical parameters associated with delaying CKD progression or lowering cardiovascular events.   **Benefits to Providers:**   - The collaborative effort between MDCT and CCN was the main key component of the intervention, where the MDCT used standard clinical guidelines to provide standard medical treatment and essential knowledge to CKD patients using live demonstration group counseling during each hospital visit, and the CCN played important roles in monitoring medication compliance, diet control, BP, and exercise behavior during a series of home visits (6–8 weeks after each hospital visit). |
| Zhang et al. [36]  2017  China | **Methodology:** Quantitative   - Interventional, cluster (town level) randomized (patient level) controlled trial, pre-post - Inpatient claim data between Jan 1, 2010, and Dec 31, 2014 (n=5,789) - **Baseline and end-point** (n=1245) in-person in-house survey and blood pressure measures from national chronic disease database (on stratified random sample of 300 patients from each town)   Main outcomes:   - systolic blood pressure - health-related quality of life ( SF36 - Medical Outcome Study Short-Form 36-Item Health Survey)   Secondary outcomes:   - hypertension-related hospitalization rate - inpatient spending   **Sample:** n=5462 hypertension patients age 35+ years from six towns, randomized by town into 3 groups (IG1: Intervention Group I; IG2: Intervention Group 2; CG: Control Group)  **Sex:**  IG1: 51% female  IG2: 54.2% female  CG: 55.9% female  **Age:**  IG1: mean age 64.5 years  IG2: mean age 66.5 years  CG: mean age 65.5 years  **Race:**  not reported | **Rural**   - *‘Six rural towns in Qianjiang county’ with an ‘average town population approximately 18,000, and each town consists of approximately 10 villages.’*   *‘Qianjiang county is highly representative of China’s rural areas, in terms of its population size, mobility, economic development state, and prevalence rates of chronic diseases.’* | **Hypertension** | **IPC:**   - multidisciplinary team-based care model   **PCPs:**  Each patient had a potential team of n=27 providers.  Village (Note that towns consist of villages)   - Clinicians (each village has 1 clinician)   Town hospitals (6); each with   - Physicians (3-4, outpatient and inpatient) - Chronic disease coordinators/educators (3) - Case Manager (1)   Public county hospitals (2)   - 20 Physicians (10 from each hospital, 5 from neurology, 5 from cardiology; 4 outpatient and 6 inpatient)     **Specialist involvement:** No | **Multi-component hypertension management.**  **Two care components:**  **Integrated care model (Aug 1 2012 to June 30 2014):** to combine treatment and prevention care with a multidisciplinary team; and to provide continuous care coordination across the village-town-county three-tier delivery system.  **Financial contracts (June 1 2013 to June 30 2014):** a contract was signed with the two towns in Group 2 where providers would receive financial bonuses if inpatient spending decreased above benchmarks. | **Challenge:**   - Hypertension management and healthcare in rural China in general was reported as fragmented across a 3-tier system (village/town/ county) with poor communication across multiple providers. | **Benefits to Patients**   - Lowered blood pressure, reduced rates of hypertension-related hospitalization   **Benefits to Providers**   - Improved interactions, communications, and document sharing, and among multiple providers across three tiers (village/town/county) - Potential financial incentive. |
| Prasad et al. [37]  2014  Canada | **Methodology:** Quantitative   - Observational, retrospective chart review   - Number of new geriatrician referrals and follow-up visits before and after program launch   - Number of NP visits in primary care, in-home, retirement home and hospital, and the number of discharges home from hospital, and length of stay   - Number of referrals to community services, and number of patients/families provided with community services info/resources, and number of patients using community services for the first time   **Sample:** Family Health Team (FHT) NP-Geri patients (no sample n’s reported)  **Age:**  84% age 75 years and older  **Sex:** not reported  **Race:** not reported | - **Rural** - **Southeast Ontario** - “farming community” | **Nonspecific ‘multiple comorbidities’ such as**  Delirium Depression  Dementia  Cognitive impairment  Congestive heart failure (CHD)  Diabetes  Chronic Obstructive Pulmonary Disease (COPD)  Osteoporosis  Gait impairment/falls  Incontinence | **IPC:**   - interprofessional care - collaboration and cross-sector care   **PCPs:**   - NP (with geriatric training) - Physicians - Nurses   **Other professionals:**   - Unspecified care providers in hospitals, LTC, and community-based services   **Specialist involvement:** Yes; Geriatrician - direct consultations and referrals | A **multi-sector, case finding and management model of care,** **Nurse practitioner (NP) – led** with primary care providers, consultation with geriatrician, and collaboration with community partners and families, with proactive visits and care provision typically in the patient’s home.  **Includes:**   - Case finding (identification of at-risk seniors), Assessment and Recommendations (episodic and chronic care), System navigation and self-management support.   **The NP is ‘the glue’ connecting existing services** that act in silos, and supports discharge planning, follow-up, and cross-sector transition, communicating via the **EMR for optimal cross-sector information sharing.** | **Challenges:**   - Potential difficulties with sustainability of this care model that **relies on using existing or even more resources** moving forward as older adult numbers increase.   **Strengths:**   - “*small cohesive team allows for easy interaction with care partners across sectors and is* ***easily adaptable to most primary care settings****, particularly in areas where geriatric-specific expertise is limited, as is the case in this predominantly* ***rural*** *area.”* | **Benefits to Patients**   - **Patient-centered**, **proactive care** provided **in-home** - *improved cross-sector care capacity and transitional support* - NP-Geri **improved access to care**, where case-finding contributed to **increase in** number of individuals who received **specialized geriatric care**, and “*trended toward more referrals from the FHT, and more follow-up visits (which stabilized over time).”* - *Even though number of admissions to complex continuing care increased, the* ***average length of stay there decreased*** *over time, especially after NP-Geri initiated weekly rounds.* - ***Percentage of patients discharged home increased*** *from 19% in 2008-09 to 31% in 2009-10, and 26% in 2011-12.*   **Benefits to Providers**  *Team integration of care between FP, NP-Geri, geriatrician.* |
| Bray, et al. [38]  2013  United States | **Methodology:** Quantitative   - Interventional, non-randomized, prospective   Baseline, 18 months, 36 months   - - Clinical measures: BP, hemoglobin, lipids/ cholesterol   **Sample:** n=727 African American patients with established type 2 diabetes randomly drawn from the larger group of patients.  Intervention Group (IG): 368,  from 3 purposefully selected rural fee-for service primary care practices.  Control Group (CG): 359, from 5 similar rural practices.  Usual care (CG) *“practices were selected by stratified randomization (ie, stratified by practice financing to match the rural health clinic and community health center funding status in the intervention practices) from a sampling frame of similar regional practices (eg, similar practice financing and payer mix) and patient characteristics (eg, race, age).”*  **Age:**  IG: mean age 59.5 years (+/12) CG: mean age 60.6 years (+/- 12.4)  **Sex:**  IG: 66% female  CG: 63% female  **Race:** African American | **Rural**   - rural counties as defined by the US Census Bureau as non-metropolitan statistical areas and population density less than 1000 persons per square mile | **Diabetes**  ‘multiple comorbidities’ | **IPC:**   - **Interprofessional care team shared across 3 practices provided** collaborative, “proactive, individualized, office-based care management and follow-up as part of the usual office visit for adult patients with type 2 diabetes mellitus”   **PCPs:**   - Physician - Nurse - Pharmacist - Dietitian care manager   **Specialist involvement:** No | **Point-of-care interprofessional team-based diabetes care management, multi-component** with education, self-management coaching, and team-based prescription (Rx) adjustment. | none reported | **Benefits to Patients**   - Improvements in reaching clinical targets - glycated hemoglobin levels, LDL and HDL cholesterol values, BP   **Benefits to Providers**   - “[intervention] strategy suggests that a portion of chronic diabetes management can be accomplished with an interprofessional team, potentially making the clinicians more available for acute problems.” |
| Fletcher et al. [39]  2012  Canada | **Methodology:** Quantitative   - Interventional, retrospective (clinical chart audit) and pre-post (baseline and 12 to 18 months at study-end)   **This study reports on the intervention arm** **only**, of the Hogg et al. 2009 RCT.   - - Medication appropriateness index   - Drug-related issues   **Sample:** n=117 patients (from the n=120 intervention arm only in RCT, Hogg et al. 2010) where n=241 were drawn from 316 patients age 50+ years contacted and identified by their family physicians as at risk for functional decline, physical deterioration, or experiencing an adverse event requiring emergency services.  **Of n=120**  **Sex:** 63 female, 57 male  **Age:** mean 69.6 years  • 50-59: n=24  • 60-69: n=33  • 70-79: n=39  • ≥ 80: n=24  **Race:** not reported | **Rural**   - Ontario   *a family health network … serving 10 000 patients in a rural area near Ottawa* | **Diabetes**  **Coronary artery disease**  **Congestive heart failure (CHF)**  **COPD**  and  comorbid chronic conditions:  • Hypertension  • Cancer  • Ischemic heart disease or atrial fibrillation  • Peripheral vascular disease  • Anemia   - Cerebrovascular disease   • Arthritis or back problems  • Asthma  • Chronic anxiety, depression, or other mental illness  • Neurologic condition | **IPC:**   - multidisciplinary collaborative team   **PCPs:**   - Family Physicians (FP) (8) - Pharmacist (1) - Nurse Practitioners (NP) (3)   Existing family health network of 8 FPs included 5 nurses, and 11 administrative personnel.  **Specialist involvement:** No | **Anticipatory and Preventive Team Care (APTCare)** – a home-based multi-disciplinary (FP, NP, pharmacist) team collaborative to improve quality of care for older adults living in the community and at risk of poor health outcomes (intervention arm only).  A pharmacist and 1 of 3 nurse practitioners visited each patient at his or her home, conducted a comprehensive medication review, and developed a tailored plan to optimize medication use. | none reported | **Benefits to Patients & Providers**  Considerable decrease in the proportion of patients that were using medications inappropriately; many patients were taking drugs that appeared not to be indicated and some were not receiving drugs they needed, which could have an important effect on health outcomes. Particularly at risk were older adults, those taking multiple medications, and those less educated. |
| Boise et al. [40]  2010  United States | **Methodology:**  Quantitative   - Interventional (pilot), nonrandomized, pre-post   **Pre-intervention:**  Chart review of patient records with clinical visits in a 2 to 4 week period in October 2006, reviewed for incident dementia Dx during following 4 months)  **During Intervention:**   - **Memory Screen** - 3 to 4 ‘possible dementia’ indicators - Patient satisfaction survey (immediately after screening)   **Pre-Post** (initial workup, and follow-ups conducted over 2 months, after end of 3-month screening period)   - **Memory Evaluation** - Memory evaluation form - Mental status exam, abstraction and judgment questions, recall, clock drawing - Verbal fluency - Activities of daily living (ADL) and independent activities of daily living (iADL) scales - Time up and go (functional mobility) - Geriatric Depression Scale (GDS) - Caregiver Burden (Zarit Burden Interview, ZBI-Short version) - Clinician dementia care confidence scale (DCCS) - Feedback sessions and surveys of clinicians and practice staff   **Sample:**  From a convenience sample of 6 rural primary care practices,  Clinicians: n=18  Medical assistants: n=26  Patients seen: n= 703, 427 that were screened, 198 of which screened positive, and 61 that completed dementia clinical evaluation.  Patient records (usual care): n=310, drawn from records of patients (age 75+) seen by study clinicians during 2 to 4 week period in October 2006 (reviewed for incident dementia diagnosis over 4 months)  **Age:** 75 years and older  **Sex:** not reported  **Race:** not reported | - **Rural** - Oregon (western) - in a ‘Rural Practice-based Research Network (ORPRN), a statewide network of 46 primary care practices in 35 communities that serve approximately 223,000 patients’. | **Dementia** | **IPC:**   - no explicit mention of interprofessional collaboration or team-based care   **PCPs:**  Eighteen clinicians and 26 medical  assistants in 6 rural practices participated in the intervention.   - 11 Family Practice Physicians - 2 Nurse Practitioners - 4 Physician assistants   **Other personnel:**   - 26 Medical assistants (classified as ‘nonclinician’)   ‘Study clinicians, medical assistants, nurses, and front office staff participated in a 2-hour training delivered via web conference by the study geriatrician and the director of the Alzheimer’s Association (Oregon Chapter) and the study primary investigator, and each site had an onsite support ORPRN staff person’ [ORPRN Practice Enhancement  and Research Coordinator (PERC)]  **Specialist Involvement:** Yes; internal medicine physician, study geriatrician | **The Rural Older Adult Memory (ROAM) Study**: A Practice-based Dementia Screening and Diagnosis Intervention over 3 to 5 months (compared to usual practice).  Authors adapted ‘materials and procedures for the dementia specific component of the Assessing Care of the Vulnerable Elders (ACOVE) model with 5 clinic-based methods to improve practice: (1) efficient collection of condition-specific clinical data, (2) medical record prompts to encourage performance of essential care processes, (3) patient education materials and activation of the patient’s role in follow-up, (4) physician decision support, and (5) physician education.’ |  | **Benefits to Patients:**   - More patients were screened for dementia, and more were diagnosed, particularly among older patients. - Most patients responded positively to having their memory evaluated, and agreed that in general memory evaluation for older adults was a good idea.   **Benefits to Caregivers:**   - One clinician quotation stated that the intervention helped get the family involved.   **Benefits to Providers:**   - Clinician confidence in diagnosing dementia improved. - Clinicians and medical assistants found the ROAM protocol easy to implement, and found the training and materials helpful |
| Hogg et al. [41]  2009  Canada | **Methodology:** Quantitative   - Interventional, randomized controlled trial, longitudinal retrospective chart audit   At baseline and 12 to 18 months study-end:  Chart audit   - - Chronic disease management score (a CDM quality of care composite score based on 12 indicator maneuvers for 4 CDs, developed as a measure of adherence to guidelines)   - Mean hemoglobin A1C and BP   - Adherence to the Canadian Task Force on Preventive Health Care recommendations for 6 preventive indicator maneuvers over previous 24 months   Questionnaires   - - Health-related quality of life scales and Short Form 36   - Instrumental Activities of Daily Living   - Self-reported emergency visits and hospitalization   - Caregiver burden   **Sample:** n=241 of 316 patients age 50+ years contacted and identified by their family physicians as at risk for functional decline, physical deterioration, or experiencing an adverse event requiring emergency services.  IG: n=120  CG: n=121  **Sex:** 159 female, 82 male  IG: 48 male  CG: 37 male  **Age:**  IG: mean 69.6 years  CG: mean 72.8 years  **Race:** not reported | **Rural**   - Ontario - *a family health network … serving 10 000 patients in a rural area near Ottawa* | **Diabetes**  **Coronary artery disease**  **CHF**  **COPD**  and  comorbid chronic conditions:  • Hypertension  • Cancer  • Ischemic heart disease or atrial fibrillation  • Peripheral vascular disease  • Anemia   - Cerebrovascular disease   • Arthritis or back problems  • Asthma  • Chronic anxiety, depression, or other mental illness  • Neurologic condition | **IPC:**   - multidisciplinary collaborative team   **PCPs:**   - Family Physicians (FP) (8) - Pharmacist (1) - Nurse Practitioners (NP) (3)   Existing family health network of 8 FPs included 5 nurses, and 11 administrative personnel.  **Specialist involvement:** No | **Anticipatory and Preventive Team Care (APTCare)** – a home-based multi-disciplinary (FP, NP, pharmacist) team collaborative to improve quality of care for older adults living in the community and at risk of poor health outcomes, compared to usual care (FP only). | none reported | **Benefits to Patients**   - Multidisciplinary care management with NPs, a pharmacist, and the family physician improved the quality of chronic care delivered to older, complex patients, particularly for those with diabetes. |
| Izquierdo et al. [42]  2007  United States | **Methodology:** Quantitative   - Interventional, randomized controlled trial, pre-post   **Baseline, and 36 months**   - - Potential adverse events/medical urgent situations   - Hemoglobin A1c (%)   **Sample:** 388 older adults from the “IDEATel (Informatics for Diabetes and Education Telemedicine Project, rural upstate New York cohort”  **Sex:** 43% male, 57% female  **Age:** mean age 71 years  (at randomization)**:**  55-64: 13.1%  65-69: 30.6%  70-74: 25.7%  75-79: 17.4%  >79: 13.1%  **Race:**  African-American (non-Hispanic) 7.1%  Hispanic 1.3%  White 91.5%  Other 0.1% | - **Rural** - **New York**   ‘**rural upstate** New York’ **‘**with **patients residing over a 30,000 square mile area**’. | **Diabetes**  (and mention of congestive heart failure) | **IPC:**   - **Collaborative team-based care** (among the diabetes care team, and then with the primary care provider).   **PCPs:**   - Nurse case managers (most were certified diabetes educators) - Dietician - Physician   **Specialist involvement:** Yes; Endocrinologist (member of diabetes team with PCPs), daily and as needed patient co-management of care. | **A 36-month team-based telemedicine intervention for patients with diabetes -** televisits with nurse case managers/diabetes educators every 4 to 6 weeks.  **Televisits conducted via a home telemedicine unit (HTU)** (video camera/ microphone), home glucose and BP monitoring devices that connect to the HTU, access to patient’s own clinical data and educational websites, and additional software. | none reported | **Benefits to Patients**   - provided access to trained/ experienced diabetes care team to patients who did not have that access previously - N=67 medically urgent events were identified and addressed |
| Bray et al. [43]  2005  United States | **Methodology:** Quantitative   - Interventional, non-randomized, pre-post   Baseline, and 12 months   - - Weight, BP, and HbA1c values abstracted from patient registry records at the intervention practice.   - Demographics and glycemic control only were collected in the control practice.   **Sample:** n=160 diabetic patients, from a convenience sample of adult patients from 2 primary care practices with an established diagnosis of type 2 diabetes  mellitus  **IG:** n=112  **CG:** n=48  **Age:**  **IG:** mean age 60 (+/- 13) years  **CG:** mean age 58 (+/- 17) years  **Sex:**  **IG:** 57% female  **CG:** 52% female  **Race:** ‘over 90%’ African American | - **Rural** - 2 rural counties in eastern North Carolina - *‘one county is designated as a federal health professions shortage area’* - Each of the two communities have approximately 3500 people and an active patient population of approximately 200 to 300 people. | **Diabetes** | **IPC:**   - interdisciplinary team - multidisciplinary team   **PCPs:**   - Physician - Nurse (Certified Nurse Specialist) - Pharmacist - Nutritionist   **Specialist involvement:** No | **Multi-component nurse-led diabetes care management** **with interdisciplinary group visit structure.**  **Components of the** intervention: care management, group visits, patient recall system, patient registry/database.  Advanced practice nurse visited  the practice **weekly for 12 months** and facilitated diabetes education, patient flow, and management. | none reported | **Benefits to Patients**   - The majority (over 60%) of patients who received the intervention had some reduction in their HbA1c, and those improvements were present across all age groups and in both men and women. - More patients achieved HbA1c goal (of less than 7%). |
| Morgan et al. [44]  2024  Canada | **Methodology:** Mixed Methods   - Observational, longitudinal, retrospective   - Semi-structured interviews (FLCs and clinic team members)   - Alzheimer Society of Saskatchewan (ASOS) First Link Client database records memory clinic client patients and family members between December 2017 and September 2022   **Sample:**  **3 FLCs and 6 team members**  from 5 rural primary health care (PHC) memory clinic teams, and **139 FLC memory clinic clients** (including persons living with dementia [PLWD], family members, caregivers).  **Sex:**  **FLCs and PHC memory clinic team members:** 100% female  **FLC clients:** 44% female  **Age:**  **FLCs and PHC memory clinic team members:** not reported  **FLC clients:** mean age at referral 69 years (mean age of PLWD at referral: 80 years)  **Race:** not reported | - **Rural** - **Saskatchewan** - Rural primary care memory clinic communities *(range 330 − 11,000 population; median 1305);*  states that: **‘***in the western Canadian prairie* *province of Saskatchewan (population 1,132,505,* *area 577,060 km2 , density 2.0 persons/km2 , 34% living* *in rural areas with less than 10,000 population)’* | **Dementia** | **IPC:**   - interprofessional collaborative care teams   **PCPs** (team composition varies depending on availability)**:**   - Family physician (FP) or Nurse practitioner (NP) - Occupational Therapist (OT) - Home care nurse (HCN) or Social Work - Physiotherapist - Dietician - Pharmacist   **Other professionals:**   - ASOS First Link Coordinator (FLC)   **Specialist involvement:** Yes; mentions specialist to provider support, with access to dementia specialists for referrals and education | **1-day interprofessional rural primary health care memory clinics led by a family physician or nurse practitioner, with an Alzheimer Society First Link Coordinator (ASOS FLC) in the role of care navigator embedded into the memory clinic care team.**  • Two new patients and their family members are seen on clinic days for a half day each. For each patient-caregiver dyad the assessment begins with a team huddle to review the referral, followed by an initial case conference with the patient and family to learn about their concerns and explain the clinic process, individual team member assessments, a team debriefing meeting, and a final team case conference with the patient and family to review the findings and recommendations. | - Both FLC and team members reported challenges with the rural location of the clinics, including **fewer services** for FLC to refer to and **travel required** for FLC to participate, and winter travel in rural areas was particularly challenging. - Limited service options reportedly made the **FLC role in the clinics seem even more important** than in larger centres, as they provide an ongoing contact and support **in the absence of other resources**. | **Benefits to FLC Clients (memory clinic patients/families)**   - **Face-to-face contact** helped establish a relationship between FLC and clients. **Compared to** FLC contact via telephone, in-person contact at the memory clinics were contacted sooner and for longer and discussed more topics. - **FLCs focus on caregivers** (whereas memory clinic team members were involved in patient assessment) and provided educational resources, emotional and psychological support, connection to services, and symptom management. - **One-day team-based model** allowed for faster, more convenient, and better overall experience. **Collaborative information sharing** among providers, with the FLC embedded into that team where the initial relationship connection with the FLC was facilitated by being in-person on clinic day.   **Benefits to Providers (Memory clinic team members) and FLCs**   - Collaborative information sharing among interprofessional team of providers. - Relief knowing caregiver needs were meeting addressed.   Learned about dementia subtypes and available services. |
| Kramer et al. [45]  2023  United States | **Methodology:** Mixed Methods   - Interventional, retrospective, pre-post   - Survey (self-assessment retrospective pre/post design; completed at the end of trainings between May 2018 and February 2020)   **Sample:** n=51 clinicians from 5 clinic sites in areas representative of diverse indigenous communities.  **Sex:** not reported  **Age:** not reported  **Race:** not reported | - **Rural** - Northwestern Plains, Southwest, Pacific Coast, and Alaska, Indian Health Service (IHS) and Tribally operated clinics *located mostly in rural areas*. | **Common geriatric syndromes including:**  Dementia  Depression  Delirium, sensory impairment, falls, gait, balance impairment , ADLs/IADLs (Activities of Daily Living, and Instrumental Activities of Daily Living), polypharmacy | **IPC:**   - Interprofessional team-based healthcare - Interdisciplinary teamwork   **PCPs:**  Referred to in the study as PCPs   - Medical Doctor - Doctor of Osteopathy - Advanced Practice Nurse (APN) - Physician Assistant (PA)   Referred to in the study as AHPs (Associate health professionals)   - Registered Nurse - Pharmacist - Rehabilitation Therapist (Physical Therapist, Occupational Therapist) - Social Worker - Registered Dietician   **Specialist involvement:** No | **IHS-RITT - an** **educational training program for primary care clinicians to improve geriatric healthcare**, based on a modified Veterans Affairs (VA) Rural Interdisciplinary Team Training (RITT) program, and is a collaboration between the VA and the IHS that supports sharing resources such as training and education.  (RITT was first implemented in 2011 to strengthen the team-based approach of patient-aligned primary care teams (PACTs; the VA’s patient-centered medical home (PCMH) model). | none reported | **Benefits to Patients**   - Prior to the training, indicators of potentially significant changes in cognition and functional impairments were not routinely recognized. - Clinicians’ were better equipped to meet the healthcare needs of older adults and persons with geriatric syndromes in the IHS and Tribal primary care settings.   **Benefits to Providers**   - Improvements to team-based care included enhanced clinical skills, organizational factors and the need to train additional employees. - Improved knowledge and ability to recognize, prioritize, and communicate effectively within an interprofessional team, and increased confidence in screening for geriatric syndromes. - Improved team-based care and individual clinical practices in assessment of cognition, function, falls, and other health and safety risks. - The intervention positively impacted clinicians ability to better meet the healthcare needs of older adults and persons with geriatric syndromes in the IHS and Tribal primary care settings. |
| Cook et al. [46]  2023  United States | **Methodology:** Mixed Methods   - Interventional, retrospective, pre-post   - **NP Satisfaction Survey** (NPSS; 28-item Likert-type scale) (residents/families)   - **Focus Group** (5 one-hour semi-structured focused interviews) (staff)   **Sample: 68 AL residents and family members (**31 residents, 29 family members, and 8 unknown) and **28 staff** from all 4 AL communities (administrative personnel, nurses or nurse assistants, medication aides, and personal care aides).  **Sex:**  Residents: 75% female  Families: 75% female  Staff: 89% female  **Age:**  Residents: 93.7% of residents were aged ≥66 years  Families: 55.4% were aged ≥66 years  Staff: not reported  **Race:**  Residents: 78.1% Caucasian  Families: 78.1% Caucasian  Staff: not reported | - **Rural** - *rural community in Northwestern United States* | **Chronic disease** (general CD management for older adults) | **IPC:**   - interdisciplinary team-based care   **PCPs**  **Main intervention delivered by:**   - Nurse Practitioner (NP) - Medical Assistant (MA) - Licensed Practical Nurse (LPN)   **Other caregivers/staff working in each AL community**   - Nurses or Nurse Assistants - Medication Aides - Personal Care Aides   **Other professionals:**   - Administrative personnel   **Specialist involvement:** No | **The NP Offsite Visit Program** is an extension of the rural health clinic in the system’s service area designed after the Optum CarePlus model, formerly known as the Evercare Model developed in 1987, where **an NP is available 3 to 5 days per week to work in consultation with patients’ PCP mainly to manage chronic disease** but additional services are also provided. On-site visits are held 1 day per week and ‘telephonic oversight’ on the remaining days. | none reported | **Benefits to Residents/Families**   - **High mean scores for all satisfaction domains** (general, communication, convenience and accessibility) (between ‘agree’ and ‘strongly agree’) - **Consistent provider(s)** allowed for more quicker, direct, one-on-one communication.   **Benefits to Staff/Providers**   - Improved communication, use of the interdisciplinary team, consistent providers, and decreased staffing needs. - All interviews appreciated the real-time conversation with the NP provider, quick assessment and treatment of acute changes, and the decrease in staff time needed to accompany residents to clinic appointments.   **Benefits to Residents/Families and Staff/Providers**   - Staff reported that the program was helpful, specifically around care coordination, preventing residents from needing acute care, and improving access to care. - Overall, staff perceived improved care, better care coordination, and improved access to care for acute needs, reducing reliance on the ED for residents’ medical needs.   Receiving care in a calm environment and timely fashion with the interdisciplinary team was beneficial. |
| Kosteniuk et al. [47]  2022  Canada | **Methodology:** Mixed Methods   - Observational, ’retrospective’ cross-sectional   - 3 post-webinar surveys and analysis of questions and comments during webinars   - **Quantitative survey data:** descriptive statistics (frequencies and proportions)   - **Qualitative questions, comments, and open-ended survey items**: were analyzed descriptively to identify themes   **Sample:** 68 rural interprofessional memory clinic team members (21/68) and other PHC professionals who attended at least 1 webinar   - 46 post-webinar surveys (29/46 respondents were memory clinic team members)   **Sex:** *‘all memory clinic team members except for one individual were female so survey respondent sex was not requested’*  **Age: (45/46 survey respondents):**   - 6 - under 30 years of age - 16 - ages 30-39 years - 13 - ages 40-49 years - 10 - ages 50-59   **Race:** not reported | - **Rural** - **Saskatchewan** - rural memory clinic teams are in **4 communities (population 300–11 000)** | **Dementia** | **IPC:**   - interdisciplinary primary care memory clinic teams - Interprofessional education (IPE) as ‘two or more professions learning with, from, and about each other to improve collaboration and health outcomes … to improve communication, teamwork, understanding of roles, and care coordination’   **PCPs:**  **Rural interdisciplinary primary care memory clinic team members** (specific composition of each team varies):   - Family physician (FP) or nurse practitioner (NP) as lead clinician - Home care nurse (HCN) - Allied health professionals (AHPs; social worker, occupational therapist, and physical therapist) - ASOS FLC   **Other professionals:**   - Administrator (executive, senior leader, manager, director) - Primary Health Care Facilitator - Alzheimer Society staff   **Specialist involvement:** Yes; education sessions led by individuals with various levels of expertise including a Geriatric Psychiatrist | **Dementia-related education: Short series (3) of webinars** delivered to interprofessional rural primary health care (PHC) memory clinic team members and other PHC professionals | **Strengths:**  **High rates of provider satisfaction** were reported for these remotely delivered education sessions, similar to the existing literature **where rural health professionals are often isolated from such dementia learning opportunities.** High rates of provider satisfaction may be related to the **remote delivery** of the sessions where perhaps rural providers’ **ease of adopting the virtual format could reflect familiarity/comfortability** with same, and led to higher rates of satisfaction. Or perhaps that opportunities for **dementia education in rural areas is lacking**, and the **need for (and appreciation of)** is reflected in the satisfaction findings.  **Challenges:**  Reported that **interprofessional memory clinic members** diverged and overlapped in specific education interests, indicating that some **may seek to broaden their knowledge** beyond traditional roles, **particularly in rural communities with fewer dementia-specific resources** | **Benefits to PCPs**  • **Overall satisfaction (90-96%) across webinars** in terms of:   - session topic/content - interactive format and webinar environment   • most (though less than 90%) intended to apply the information in their practice and appreciated the participation of other PHC teams and professionals  • rural PHC memory clinic team members have the **opportunity to:**   - **learn not only from specialist presenters, but from each other** working in similar roles, during interactive Q & A   **suggest topics for future education webinars** based on their professional wants/needs |
| Lall et al. [48]  2020  India | **Methodology:** Mixed Methods   - Observational, prospective cohort, multi-‘case study approach’   **Quantitative** (proportions and incidence by person-months in care):   - - Total n patients attended follow-up   - Average fasting blood glucose (per person-months of follow-up)   - Average BP (per person-months of follow-up)   and   - - **Process Indicators** for n patients with:     - Completed risk assessment     - BP measure     - Fasting blood glucose measure     - Foot exam     - Counseling     - ASHA home-visits   **Qualitative** (inductive and deductive analysis)**:**   - - **n=9** in-depth semi-structured **interviews**   - field notes, observations at PHC visits   **Sample:** 279 patients with diabetes, hypertension, or both, from 3 rural PHCs (PHC 1 n=36, **PHC 2 n=212**, PHC 3 n=31)  ***Interviews and outcome assessments for PHC 2 only***  **Sex: 60% female**   - PHC 1: 60% female - **PHC 2: 62.7% female** - PHC 3: 58% female   **Age:** mean age 62 (+/- 10) years   - PHC 1: 59.3 (+/- 10.1) - **PHC 2: 61.5 (+/- 10.9)** - PHC 3: 64.5 (+/- 9.1)   **Race:** not reported | - **Rural** - **Karnataka** - *‘3 publicly funded primary PHCs’* in ***‘rural South India’,*** *‘Kolar district of Karnataka state’* | **Diabetes, and**  **Hypertension**  CVD | **IPC:**   - team-based care   **PCPs:**   - Physician - Nurse(s) - Pharmacist - Community health workers (known as accredited social health activists (ASHAs)   **Other professionals:**   - Coordinator/Navigator - Lab tech   **Specialist involvement:** No | **Multicomponent* team-based care intervention, with a participatory approach** (co-designed with implementers), **and** designed and evaluated within a **theory of change framework**.  ***4 intervention** **components:** to  (1) optimization of workflow to **include essential tasks** **at each visit** (such as counselling and BP/blood glucose measurements); (2) **distribution of clinical tasks shared** among team; (3) initiate a **clinical record** for patients with diabetes or  hypertension to be retained at the healthcare facility and **retrieved at each visit** for follow- up after enrolment; and (4) **build staff** **capacity, and involve ASHAs in patient follow-up.** | **Challenges:**   - Instability of staff due to other PHC shortages - Time constraints due to staff shortages and conflicting workloads - Lack of patient involvement in planning and designing study due to ‘local cultural influences’ (staff at PHCs were not keen to involve patients or a community representative). | **Benefits to Patients**  **Outcomes at PHC 2**   - There was a **decline in both the average fasting blood glucose, and systolic BP**, **with an increase in person-months of follow-up**   **Patient feedback:**   - Among patients who attended more than 3 follow-up visits, **most viewed changes positively** - Patients identified **changes in the workflow and the clinical record as the most tangible** changes compared to previous/usual care; and most felt the **changes in workflow** **increased efficiently of the clinic consult** - Most patients reported feeling like they achieved **better control of their conditions**.   **Benefits to Providers**  Distribution of and sharing tasks, although some providers preferred to work in silos |
| Bonney et al. [49]  2017  Australia | **Methodology:** Mixed Methods   - Interventional (pilot), randomized, pre-post   - Semi-structured interviews (thematic analysis)   n=5 GPRs, pre-post educational outcomes,  n=7 GPs & Nurses, trial practicalities  n=5 control and n=6 intervention patients, pre-post, satisfaction   - - Pre-post changes in patient mean clinical parameters (BMI, blood sugar, BP, cholesterol, triglycerides)   **Sample:** from 2 rural training practices   - **Patients:** 30 (n=23 pre-interviews, 11 post-interviews [5 controls, 6 intervention, randomized within each practice]) - **Providers:**   2 GPs  5 Practice Nurses  5 GPRs (pre-post interviews)  **Sex:**  **Patients** – IG: 8 male, 6 female; CG, 8 female, 8 male  **GPs** – 2 male  **Practice nurses** – 5 female  **GPRs** – 1 male, 4 female  **Age:** mean age 71.4 years (IG 72, CG 70.8)  **Race:** not reported | - **Rural** - **Australia** - Participants were recruited from ***‘two*** ***rural training practices****’* | **Diabetes** | **IPC:**   - shared continuity - team visits   **PCPs:**   - GPs (General Practitioners) - GPRs (future GPs) - Practice Nurses   **Specialist involvement:** No | **An educational model of supervised, team-based primary care with shared chronic disease management** of rural type 2 diabetes patients between regular GP and GPR/practice nurse team compared to usual care (GP only) in two rural training practices over 8 months. | none reported | **Benefits to PCPs**   - GPRs: improved self-reported exposure to and confidence in CDM. - overall receptive to procedures and saw benefits |
| Sorocco et al. [50]  2013  United States | **Methodology:** Mixed Methods   - Observational, longitudinal, prospective multi-case study   Daily:   - - Vitals and symptoms   At baseline, 3 months, and 6 months:   - - ‘OT evaluations of physical strength’   - Self-reported social functioning   - Zarit Caregiver Burden Index (ZBI)   - Treatment compliance   - Satisfaction and interview data     **Sample:** **6 Veterans** with “complex medical conditions” **and their caregivers** living in rural Oklahoma were enrolled  Veterans  **Age:** mean age 71.83 years (+/- 10.6 years)  **Sex:** not reported  **Race:** not reported  Caregiver age, sex, race not reported | - **Rural** - **Oklahoma** - “underserved rural” | **‘a range of physical health conditions including’**  Depression  Dementia  Anxiety  Post-traumatic Stress Disorder (PTSD)  COPD  Cerebrovascular accident  Diabetes Hypertension Spinal cord injury Syncope | **IPC:**   - Interdisciplinary team   **PCPs:**  HBPC team involved in program implementation:   - Physician - Physician assistant - Nurse or NP - Pharmacist - Nutritionist - OT - Social Worker - Mental health practitioner (see psychologist mention re: specialist below)   Other unspecified staff involved in the program:   - Care Coordination Home Telehealth (and telemental health) (CCHT) - Geriatrics - Extended Care Services   **Specialist involvement:** No; psychologist-led intervention but not a specialist in this case; psychotherapy sessions, technical support | **Simultaneous involvement in both the CCHT (Care Coordination Home Telehealth and Telemental health) and HBPC (Home Based Primary Care)**, receiving same standard care as other HBPC patients but with the addition of home telehealth equipment and clinical video technology that allowed for daily remote monitoring of vitals and symptoms, and delivery of interactive televideo sessions including telemental services (psychologist) and other unique home-based services such as OT-led exercise sessions. | **Rural/Remote Challenges impacting innovation:**   - Additional equipment in rural settings was required such as surge protectors to protect equipment - Participants had to learn how to reset the equipment if needed (or would result in provider home-visit tech support)   **Rural/Remote Strengths impacting innovation:**  None | **Benefits to Patients & Caregivers:**   - Previously underserved rural veterans with complex medical conditions were served, with comprehensive care, including telemental health services (including rural veterans that were not eligible for HBPC because they lived too far away and out of the service area) - Over 6 months, improvements in physical strength, social functioning, Tx compliance, and caregiver burden - One participant reported an increase in his accountability, reduced A1C, and successful support of Tx goals with other disciplines   **Benefits to PCPs**   - **Ideally there would be less frequent in-home visits** for Tx such as home-based OT-led exercises and psychologist-led psychotherapy **however, a lack of staff dedicated to tech support counterbalanced this goal** (across all veterans in HBPC over 1 year averaged 29 visits; n=6 in this study over 6 months averaged 23.75 +/- 7.93) |
| Schubert et al. [51]  2024  United States | **Methodology:** Qualitative   - Observational, longitudinal, retrospective   - periodic reflection notes (staff involved in design and implementation, facilitated by the telehealth technician)   - fieldnotes (staff involved in implementation of hybrid-virtual home visits)   - team meeting notes (Tele-GRACE team)   - guided interviews (conducted quarterly with 5 VA-GRACE team members)   **Sample:** n=5 TeleGRACE and VA-GRACE team members (geriatrician, telehealth technician, 2 project coordinators, and a primary care provider/health services investigator).  **Ages:** not reported  **Sex:** not reported  **Race:** not reported | - **Rural** - *rural dwelling* - *more than 60 miles from the [closest] VA hospital* | **Common geriatric syndromes** | **IPC:**   - Multidisciplinary team care   **PCPs:**   - Nurse Practitioners - Social Workers - Pharmacist   **Specialist involvement:** Yes**;**  Geriatrician (facilitator), psychologist review cases and assist with developing a care plan. | **TeleGRACE: a hybrid-virtual home visit intervention** to expand access to comprehensive geriatric evaluation via telehealth **where a telehealth technician travelled to patients’ homes** and connected virtually to VA-GRACE team members who participated remotely. TeleGRACE is **a single modification of the existing VA-GRACE (Geriatric Resources for Assessment and Care of Elders) program.**  **The VA-GRACE** (Veteran Affairs-  Geriatric Resources for Assessment and Care of Elders) **Program** is a multidisciplinary care model which provides home-based geriatric evaluation and management for older Veterans residing within a 20-mile drive radius from the hospital. | - **Poor cellular and internet connectivity** in many rural areas. The telehealth technician had multiple devices on-hand (IPAD, laptop, VA cell phone), each of which used a different cellular carrier, to connect remotely with the VA-GRACE dyads.   **Travel logistics** in rural areas – for example, no ready access to safety services while enroute, and directional issues with mapping services. | **Benefits to Patients**   - Older rural VA patients living at greater distances from a VA medical center received multidisciplinary care virtually via TeleGRACE without having to leave their home, with the assistance of a telehealth technologist.   **Benefits to Providers**   - VA-GRACE supports primary care staff by offering geriatrics clinical expertise for patients who may have geriatric syndromes but do not require intensive geriatric care.   TeleGRACE allows healthcare providers to extend their reach to deliver longitudinal, multidisciplinary geriatric care in the outpatient setting to patients who reside beyond the 20 mile radius of the in-person program. |
| Bundy et al. [52]  2022  United States | **Methodology:** Qualitative   - Observational, multiple case-study approach   - Phone interviews (thematic analysis)   **Sample:** n=20  10 patient/caregiver dyads receiving virtual memory care at two rural primary care clinics between August 2020 and January 2021.  **Ages:**  Patients: 74 to 87 years (median 79.5)  Caregivers: 51 to 83 years (median 73)  **Sex:** not reported  **Race:** not reported | - **Rural** - North Carolina | **Dementia** | **IPC:**   - multidisciplinary team   **PCPs:**  States that patients were provided with “an assessment from a geriatrician, access to a dementia navigator/social worker, and follow-up care”.  As in See Liu et al. [21] for further details including:   - Dementia navigator/social worker - Initial referral by PCP (not specified; on-site) - Medical Assistant (on-site) - Social Worker (Dementia Navigator) (off-site; virtual and via phone)   **Specialist involvement:** Yes; Geriatrician (virtual) | **Virtual Memory Clinic** – “in-clinic virtual care” – practice-based virtual dementia screening and referral program using telehealth, with patient assessment by geriatrician, access to dementia navigator/social worker, and follow-up care **embedded into the primary care workflow and electronic medical record (EMR).** |  | **Benefits to Patients and Providers:**   - In-clinic appointments provided a privacy that was not guaranteed at home.   **Benefits to Patients and Caregivers:**   - In-person staff could serve as mediating presences at appointments, able to negotiate caregiver/patient relationships.   **Benefits to Caregivers:**   - Clinic-based care relieved older rural caregivers who were often less capable of, or equipped to, navigate virtual technology. - Many caregivers preferred the openness allowed for with the in-clinic format.   **Benefits to Providers:**   - In-clinic appointments provided a privacy that was not guaranteed at home. |
| Morgan et al. [53]  2022  Canada | **Methodology:** Qualitative   - Observational, retrospective, cross-sectional   - 6 focus groups, and   - 16 workgroup meetings, held with teams over **1-year post-implementation**   **Sample: rural PHC providers**, involved in designing and delivering rural memory clinics, from 4 rural primary care memory clinic teams   - **38 unique individuals** were involved in both focus group and workgroup meetings   - Focus Groups (6): 40 participants   - Workgroup Meetings (16): 100 participants   **Sex:** *‘All participants were*  *female except for two males, from two different teams,*  *who participated in the workgroups only. Focus group participants were all female.’*  **Age:** not reported  **Race:** not reported | - **Rural** - **Saskatchewan** - **4 communities (population 300–11 000)**; states that *clinics are in the prairie province of Saskatchewan in western Canada (population 1 million, area 651,000 km2 , density 1.9 persons/km2)* and that *‘39% the province’s population lives in rural areas with less than 10,000 population, compared to 19% in the rest of Canada* | **Dementia** | **IPC:**   - interdisciplinary primary care memory clinic team   **PCPs** (specific composition of each team varies)**:**   - Family physician (FP) or nurse practitioner (NP) - Occupational Therapist - Home care nurse (HCN) - Social Work - Physiotherapist - Dietitian   **Other professionals:**   - ASOS First Link Coordinator - PHC Facilitator - Manager - MOA/Office Staff   **Specialist involvement:** Yes; mentions specialist to provider support, with access to dementia specialists for consults, referrals, and education sessions | **1-day interprofessional rural primary healthcare memory clinics:**   - ‘**an intervention** that operationalized key components of best practices identified in existing literature in ways that were **feasible, effective, and sustainable in rural contexts**’ into **‘an interdisciplinary model of dementia care’** that **‘resulted in a one-stop interdisciplinary primary care-based memory clinic** providing diagnosis and post-diagnostic support for rural people with dementia and their caregivers   2 patients/families are assessed at 1-day clinics held every 1–2 months in the local PHC clinic or hospital’ | **Challenges:**  **• Limited resources where** expanding the memory clinics requires identifying potential new PHC teams that want to do it and also have capacity in personnel, time, and space.  **• Teams looked to physicians and NPs to lead clinics, but the difficulties of recruiting and**  **retaining physicians in rural communities was a barrier**  to both sustaining and scaling. **Engaging physicians was a challenge**, perhaps because  they were uncertain about how long they would stay in the community.  In the current study **turnover was a challenge** because consistency of members helped build team cohesiveness  and reduced disruption to clinic operations.  **• With high workload demands faced by rural healthcare providers**, this study suggests that **ongoing support from a designated individual with authority and responsibility for this role will be key** to sustainability. With the lack of time and competing priorities, **support was needed from team facilitators, managers, or researchers to follow up or help with execution.** | **Benefits to Patients/Caregivers**   - **rural memory clinics address gaps and meet needs** in communities (aging communities with limited services/traveling often long distances multiple times to access services); providers reported receiving positive feedback from patients and families - provide **“one-stop shop”** with access to all services and provide “wrap-around” support with an **interprofessional, collaborative team-based approach** - **community awareness that help is available** leads to continued referrals - **patients/families more comfortable with familiar PHC team** members - **avoid travel/wait times** for urban specialists - **families expressed relief at getting help and less alone and isolated** because they know where to go for help; **facilitated introduction to Alzheimer Society** First Link Coordinator and their available services and supports - clinics are resulting in **earlier diagnosis and management**   **Benefits to PCPs**   - Teams want to implement and sustain **and valued being able to provide service where previously they had little to offer** - **team members are more comfortable with each other and working together** within an interprofessional team-based approach - **collaborations have spread** to work **outside clinic** - **team members are more aware of each other’s practice and contributions** to dementia care - **team members have more information to work with**, improving individual/joint recommendations - **Alzheimer Society can establish relationship with patient and family** to discuss needs and supports - clinics are **efficient** for some team members **because assessments and planning are coordinated in one visit** - **team approach is gold standard;** takes pressure off individual members for issues such as driving - memory clinic has core components but is **adaptable** depending on available health care professionals - **everything needed is ready to use** so less daunting to start a clinic, and less time investment required by teams than trying to start a memory clinic on their own - **standardized processes and tools** (PC-DATA flowsheets in EMR, RaDAR Handbook, etc.) provide structure to follow and reduces uncertainty about what is expected **education sessions,** **shadowing/mentoring** opportunities **build capacity and confidence** |
| Li et al. [54]  2020  China | **Methodology:** Qualitative   - Observational, retrospective cohort   - 5 focus groups (content analysis; normalization process theory)   **Sample:** 38 care team members (6 psychiatrists, and 2 cohorts with 8 village doctors and 8 aging workers in each) that participated in COACH RCT  **Age:** mean age 42 years  **Sex:** 28 female, 10 male  **Race:** not reported | - **Rural** - **Multiple villages in rural China** - Describes **rural China** as a **low resource setting** where **village clinics** provide primary care and travel to access secondary and tertiary care is limited due to **transportation barriers** | **Comorbid Hypertension and Depression** | **IPC:**   - collaborative - multidisciplinary team’   **PCPs:**   - Village Doctors (VDs) - Aging Workers (AWs) (‘Women workers’ and ‘Health liaisons’)   **Specialist involvement:** Yes; Psychiatrist Consultants - psychiatrists from the county mental hospital provide consults to VDs when required, and travel to villages to conduct diagnostic assessment and in consultation with the VD to initiate antidepressant Rx when needed | **Chinese Older Adult Collaborations in Health (COACH) intervention12-month team-based collaborative care intervention** to treat older rural adults with co-morbid hypertension and depression. | **Strengths:**   - VDs and AHs typically live and work in the same village; their **familiarity with each other, their patients, and their village** enhanced communication and collaboration; VDs and AWs genuinely care about their village and older adults and want to help, which appeared to help sustain their commitment to the intervention - **Organizational structure** **and support of leadership**, with village clinics (PCPs) under county hospital (psychiatrist) administration supported team member collaboration, facilitated recruitment, and engagement due to perceived leadership-level buy-in   **Challenges:**   - **Extensive psychiatrist travel time** to attend villages for Dx assessments inevitably negatively impacts workload, though only reported by one | **Benefits to Patients**   - AWs were seen less as a medical professional and **helped decrease older adults’ resistance to mental health care** - AWs reported that they were able to establish **trusting relationships with patients through continuous contact and small talk**, and patients would confide in them - AWs felt **their home visits contributed to patient improvements** in mood, which led to improvements in their health - VDs and AWs felt **family support increased** as a result of the intervention, due to both the **reduced depressive symptoms** and the family education that **improved communication**   **Benefits to PCPs**   - **Integrated behavioral health and medical care** allowed for each team member to bring their individual professional knowledge and strengths to the table which facilitated a ‘whole-person understanding of the patient’ - **VDs** perceived the **integration of hypertension and depression** **management** as **efficient and effective** - **All team members** appraised COACH model positively and reported positive changes in patients and the reciprocal relationship of mood and hypertension - **High level of team interaction** with joint planning and problem-solving - **Intervention activities** were **skill-level appropriate** to team member role and **easily integrated** into regular practice - **Training sessions** helped solidify engagement, enhance knowledge, and facilitate collaboration overall |
| Morgan et al. [55]  2019  Canada | **Methodology:** Qualitative   - Observational, longitudinal retrospective (process evaluation over 2.5 years) - Focus groups (4) - Workgroups (3) - Phone interviews (4) - *‘Data collection and analysis were guided by the Consolidated Framework for Implementation Research (CFIR) which consists of 38 constructs within five domains.’ (deductive analysis)* - *Inductive analysis*   **Sample: n=25 unique individuals**  PHC team members (13), Managers (8), Office staff (4)  **Age:** not applicable  **Sex:** 23 female, 2 male  **Race:** not reported | - **Rural** - Saskatchewan - *‘population 1 million, area of 651,000 km^2^’* - ‘Sun Country Health Region population 60,000 area 33,329 km^2^, density 1.8 persons/km^2^,’ and ‘two urban centres of approximately 11,000 people, with 58% of the population living in rural areas under 10,000 population.’ | **Dementia** | **IPC:**   - interdisciplinary team-based care   **PCPs:**   - 3 Family Physicians - Nurse Practitioner - Occupational Therapist (OT) & Regional Manager of Therapies - 2 OTs   **Other professionals:**   - Alzheimer Society First Link Coordinator - 2 PHC Facilitators - PHC Regional Business Manager - 4 office staff - Home Care Manager - Manager Home Services - Alzheimer Society Manager - Community Health Services Manager - CD Management Coordinator - 3 Regional Managers (PHC Teams, CD Management, and Mental Health and Addictions)   **Specialist involvement:** Yes; specialist-to-provider support was provided by research team members with regular education sessions (both in-person and via telehealth) | **Interdisciplinary rural primary health care 1-day memory clinic.**  An evidence-based intervention in local rural setting, developed and implemented using a collaborative community-based participatory approach.  “The memory clinic, which is ongoing, is held every one to 2 months, with two patients and their families attending for a half-day each. “The 1-day memory clinic involves a team huddle to review the concerns leading to the referral and any previous testing, a team case conference with the patient and family, individual team members’ assessments, a team debriefing, and a final team case conference to discuss the findings with the patient and family and develop a care plan. Team-based follow-up appointments scheduled at three to 6 months. Evidence-based decision support tools are used to guide the initial evaluation and follow-up appointments.” | **Challenges:**   - Turnover, recruitment, and retention of PHC team members (only 2 original team members were still with the team at the end of the study). - “Lack of co-location of PHC team members made it more difficult and time-consuming for them to meet and limited the opportunities for informal conversations”, as did the long distance travelled by researchers to the rural PHC team community.   “The RaDAR team’s 20-year experience in rural dementia research, and evidenced in the current study, is that rural health care providers and those involved in planning services are resourceful, collaborative, engaged with their community, and innovative in addressing community needs. The PHC team’s commitment to the long iterative process of co-designing and implementing the rural PHC intervention indicates their commitment to improving practice gaps for people with dementia and their families, despite the many challenges.” | **Benefits to Patients**   - Team members reported that “The coordinated team approach facilitated discussions about available services and planning for future needs” allowing for earlier diagnosis and support, avoiding potential crisis situation and long-term care placement.   **Benefits to Family**   - “Team members identified benefits to families, which included giving them a voice, providing direction, and enabling them to plan for the future and avoid crises. Team members had seen the negative consequences of not connecting patients and families with available supports early on and were relieved that this gap was filled by Alzheimer Society First Link coordinator with specific skills in assessing and supporting patients and families.” - “Team members anticipated that by including the Alzheimer Society First Link Coordinator in the clinic assessment the family would be more likely to accept supports in the future.”   **Benefits to Providers**   - “Team members reported that the team-based standardized evidence-based assessment flow sheets helped them provide better care by providing a template or guide to assessment steps. They reported increased confidence and feeling empowered to provide better care without having to refer all patients to specialists.” - “The team approach also allowed team members to contribute their individual disciplinary skills to the assessment. They felt valued by other team members and in turn appreciated other team members’ roles. The format of the one-day clinic allowed team members to discuss their findings and learn from each other, thus increasing their confidence in the diagnosis and treatment plan. The synergies of working together resulted in better care, which was rewarding.” - “Ability to test the innovation on a small scale and undo the implementation if needed was important because it allowed the team to iteratively develop, test, and modify strategies to implement team-based care and the decision support tools to fit their context.” - Having the PC-DATA flowsheets integrated into the EMR “was critical because the EMR was standard practice in the clinic and the flow sheets helped to operationalize several components of the RaDAR PHC Model, including coordinated team-based care and access to evidence-based decision support tools. The EMR also allowed functions such as ability to download and print embedded scales, and links to resources and referral forms.” - “Most team members supported the project because they had long-standing concerns about unmet needs and believed that the intervention could help with earlier diagnosis and maintaining the person with dementia in the community.” - “Education about dementia assessment and management were important in building team members’ confidence in conducting the memory clinics.” |
| Wong et al. [56]  2015  Canada | **Methodology:** Qualitative   - Observational, multiple case-study   - In-depth interviews and direct observation (interpretive thematic analysis)   **Sample:**  From 5 PHC practices with GMVs in rural towns and four First Nations communities  **34 Providers**   - 10 FPs, 7 Nurses, 2 NPs, 4 PHC Coordinators, 11 Others (MOA, CHR, Outreach Coordinator, etc.)   **29 Patients**  **Age:** mean 62 (+/- 16) years  **Sex:** 65.52% female  **Race:**  55.17% European (Caucasian)  41.38% First Nation  3.45% Metis | - **Rural** - **British Columbia** - **Northern Health Authority** *‘communities ranged in population size from 200 to 76,000; some communities were up to 1,000 km away from Prince George, BC’s northern regional city’* | **Nonspecific**  Most commonly reported: diabetes, hypertension, arthritis  Also reported: depression, heart disease, kidney disease, cholesterol, other | **IPC:**   - Interprofessional teams - collaborative team-based care   **PCPs:**   - Physicians - Nurse Practitioners - Nurses   **Other professionals:**   - PHC Coordinators - MOA - Community Health Representative - Outreach Coordinator   **Specialist Involvement:** No; coordination of referral only | **Group Medical Visits (GMV) in primary health care.** ‘GMVs are visits with one’s regular provider that take place in a group of 10–15 patients … meant to increase timely access to primary care [drop-in type], and the quality and comprehensiveness of services delivered to patients who share common healthcare needs, and facilitate increased self-management of an individual’s health. Other healthcare providers and community organizations (e.g., local recreation centre) are sometimes present’ | none reported | **Benefits to Patients & Providers**   - Regularly held (every 1 to 2 months) GMVs **fostered access** to needed primary health care services for all patients where some providers were able to accept new patients due to GMVs’ **increased the overall capacity of the practice**, gaining time from not going from room to room, grouped patients, etc. and decreased patient waiting time - GMVs also **increased opportunity for preventive practices** (such as vaccinations and disease screenings) **and health promotion activities.** - Synchronous communication **between providers, and provider-patient** allowed for easy adjustments in Tx plans or follow-up and education opportunities. - **Expanded opportunities for collaboration and team-based care** between *healthcare providers and public health (e.g., public health nurses), the Health Authority, and healthcare providers working in first Nation communities such as Community Health Representatives.* - *Direct observation revealed that* ***team members and patients*** *were working together to find potential solutions for individuals’ medically and socially complex care needs.* - *Across all Gmvs,* ***patients became actively involved in a team-based care approach.*** *Patients reported* ***learning from other health professionals*** *such as dieticians* ***and their peers*** - Improved **both patient and provider** experiences where p*atients reported increased confidence and skills in managing their health, and were motivated by knowing they were not alone* - *GMV structure helped neutralize inherent* ***patient-provider*** *power imbalance* - ***Providers*** *reported that attending GMVs could lead to improved clinical indicators, and found the GMV environment an easy one to deliver comprehensive care for those with complex care needs* |
| Tolson et al. [57]  2007  United Kingdom | **Methodology:** Qualitative   - Observational, formative evaluation (Realist approach), multiple case pilot study   Implementation and progress review and evaluation at 6 months, 11 months, and 15 months.   - - Semi-structured stakeholder interviews (patients, family carers, direct care health care professionals)   - Focus groups (3) (Network Executive members)   - Document review (flip cart notes/diagrams, individual notes, field notes)   **Sample: 3 older men, their families, the doctors and nurses providing direct care, and**  **13 members of the network management group.**  n=3 palliative patients with cancer-related pain, their 4 family members), health care providers directly involved in their care (GPs, district nurses, specialist nurses), and 13 network executive members (health care professionals representing General Practice, District Nursing, Specialist Palliative Care Medicine/Nursing, and Pharmacy)  ***Where reported, participant sex/gender, ages, and race, by case:***  **Case study 1 (Timepoint 1 – 6m; one general practice)**  *Interviews:*   - Patient (male, ‘in his 70s’) - Spouse (female) - Daughter (female) - 3 district nurses - 1 specialist nurse - General Practitioner (GP) *Focus Group:* - 6 Network Executive Group members   **Case study 2 (Timepoint 2 – 11m; 3 general practices)**  *Interviews:*   - Patient (male, ‘in his 70s’) - Wife - District nurse - Specialist nurse - General Practitioner (GP)   *Focus Group:*   - 8 Network Executive Group members   **Case study 3 (Timepoint 2 – 15m; 4 general practices)**  *Interviews:*   - Patient (male, ‘elderly’) - Wife - District nurse - General Practitioner (GP)   *Focus Group:*   - 11 Network Executive Group members | **Rural**   - rural Scotland | **Cancer**  Depression   - Case 1: **esophageal cancer** - Case 2: **squamous cell carcinoma** (of lung, and metastases) - Case 3: **prostatic cancer** (with widespread bony metastases) | **IPC:**   - Coordinated, collaborative approach to interdisciplinary team-based care   **PCPs:**   - District nurses (3) - General Practitioners (GPs) (3) - Pharmacists (for Cases 2 & 3)   **Other professionals involved:**   - Network Executive members (13), a multidisciplinary group of health professionals representing General Practice, District Nursing, Specialist Palliative Care Medicine/ Nursing, and Pharmacy - Practice development facilitator   **Specialist involvement:** Yes; specialist nurse (cases 1 & 2) | **To improve cancer-related pain management,** a new **interdisciplinary managed care network (MCN)** approach (implemented over 18 months) with focus on context-mechanisms-outcomes (CMOs); context being workplace challenges, and mechanism being facilitation processes, involved in positive/negative feedback) to develop the most effective practice model to implement the Scottish Intercollegiate Guideline Network  SIGN 44 guidelines for pain management.    **MCNs were described as** linked groups of health professionals and organizations from primary, secondary and tertiary care, working in a coordinated manner, unconstrained by existing professional and service boundaries. | none reported | **Benefits to Patients**   - Patient accounts of positive relationship with district nurses. - Patients (and families) found pharmacist helpful, and in general reported better pain control.   **Benefits to Providers**   - District nurse accounts of increased job satisfaction. - District nurses greater involvement in medication management perceived by nurses to as strengthening patient trust. |
| Keady et al. [58]  2004  United Kingdom | **Methodology:** Qualitative   - Interventional, single case history - Case history report based largely on detailed notes of the community mental health nurse delivering the intervention   **Sample:** 1 person living with dementia and 1 family carer, from a sample pool of n=50 people living with dementia and their family carers that participated in this project (23 assigned to IG, 27 to CG), recruited by identification through a previous community study (20), and from those identified in a primary care dementia screening program (30).  **Age:** patient and caregiver > 75 years  **Sex:** male person living with dementia, female family carer  **Race:** not reported | - **Rural** - North Wales - *‘small village’* | **Dementia** | **IPC:**   - ‘A number of experienced professionals from different professional backgrounds’ were involved, and close liaison with the primary care team   **PCPs:**   - GP - Community mental health nurses (2) - Psychiatric social worker   **Other professionals:**   - Project manager   **Specialist involvement:** Yes; clinical psychologist provided intervention training to, and clinical supervision sessions with, nurse and social worker | A single case history of the **Dementia Action Research and Education project** – a **15 month** primary care intervention in dementia care consisting of early and psychosocial interventions with older people living with dementia and their families, and regular in-home follow-up visits.  “…interventions were individualized, person and family centred” | none reported | **Benefits to Patient**   - GP attended patient’s home to communicate the Dx ‘honestly, clearly, and sensitively’. - Patient was able to understand the Dx and be involved in developing more personalized disease management strategies. - Patient developed trust and confidence in their provider and was comfortable being open about issues, fears, etc., and felt understood and empowered. - Patient grew more comfortable sharing Dx and communicating needs. - Patient was able to continue living in own home with appropriate supports.   **Benefits to Family Carer**   - Able to explore concerns in advance about sharing Dx, reducing related fears and anxiety, and felt able to be more open and honest.   **Benefits to Providers**   - Community mental health nurse developed a good understanding of patient and family carer needs and offered meaningful, in-home support, including early identification of any coping issues. |
